# Investigating Neuron Degeneration in Huntington’s Disease Using RNA-Seq Based Transcriptome Study

**DOI:** 10.3390/genes14091801

**Published:** 2023-09-14

**Authors:** Nela Pragathi Sneha, S. Akila Parvathy Dharshini, Y.-h. Taguchi, M. Michael Gromiha

**Affiliations:** 1 Department of Biotechnology, Bhupat and Jyoti Mehta School of Biosciences, Indian Institute of Technology Madras, Chennai 600036, Tamil Nadu, India; pragathi.sneha91@gmail.com (N.P.S.); akilabioinfo@gmail.com (S.A.P.D.); 2 Department of Physics, Chuo University, Kasuga, Bunkyo-ku, Tokyo 112-8551, Japan; tag@granular.com

**Keywords:** Huntington’s disease, Brodmann area 4, tissue-specific network analysis, variant effect, function interaction network

## Abstract

Huntington’s disease (HD) is a progressive neurodegenerative disorder caused due to a CAG repeat expansion in the huntingtin (*HTT*) gene. The primary symptoms of HD include motor dysfunction such as chorea, dystonia, and involuntary movements. The primary motor cortex (BA4) is the key brain region responsible for executing motor/movement activities. Investigating patient and control samples from the BA4 region will provide a deeper understanding of the genes responsible for neuron degeneration and help to identify potential markers. Previous studies have focused on overall differential gene expression and associated biological functions. In this study, we illustrate the relationship between variants and differentially expressed genes/transcripts. We identified variants and their associated genes along with the quantification of genes and transcripts. We also predicted the effect of variants on various regulatory activities and found that many variants are regulating gene expression. Variants affecting miRNA and its targets are also highlighted in our study. Co-expression network studies revealed the role of novel genes. Function interaction network analysis unveiled the importance of genes involved in vesicle-mediated transport. From this unified approach, we propose that genes expressed in immune cells are crucial for reducing neuron death in HD.

## 1. Introduction

**Figure 1:**
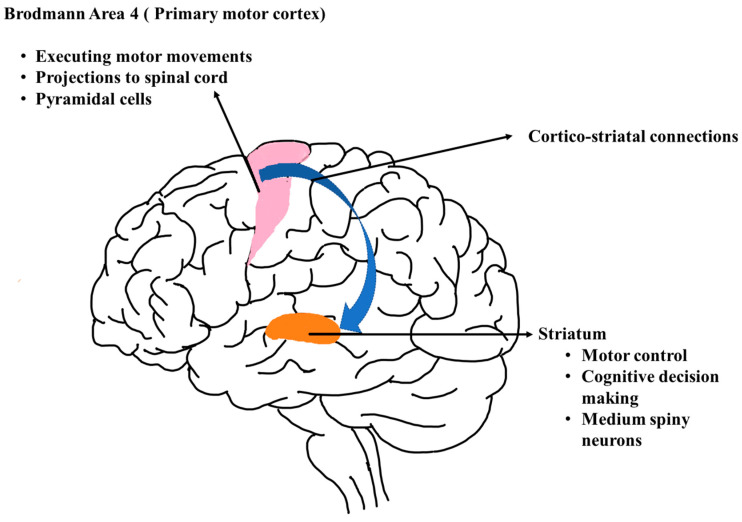
Simplified diagram of the brain with illustrations of regions affected in Huntington’s disease along with their functional role.

Huntington’s disease (HD) is a trinucleotide repeat disorder caused by a CAG repeat expansion affecting the medium spiny neurons in the brain [1,2,3,4]. CAG repeat expansion is caused by the *HTT* gene, which encodes for the huntingtin protein [5,6,7]. HD is a serious neurodegenerative disorder with motor and cognitive symptoms such as chorea and memory loss, respectively. Patients with this autosomal dominant disorder are susceptible to motor dysfunction, cognitive deficits, and behavioral changes. The only FDA-approved drug to treat HD-associated chorea is Tetrabenazine [8], and other treatments include ingestion of anti-psychotic drugs such as haloperidol and pimozide. On the other hand, donepezil is used for cognitive dysfunction and fluoxetine for depression, and surgical treatments including deep brain stimulation are used [9], which are palliative and not disease-modifying. HD affects the striatum in basal ganglia by degenerating medium spiny projection neurons (striatal neurons). The basal ganglia are responsible for voluntary movements, while major input comes from the striatum for coordinating motor activities. Striatal neurons are more vulnerable in the process of neurodegeneration in HD [10], whereas the motor cortex is the core region of the brain regulating movement. The connections between the striatum and motor cortex are prominent and are essential for coordinating locomotion and behavioral variability. Brodmann area 4 (Figure 1) of the motor cortex is the anchor for initiating voluntary movement, and its projections to the striatum are crucial for cortico–striatal communication, which is responsible for momentary action [11,12,13]. HD symptoms are mainly related to locomotion/movement. Exploring gene and transcript-level expression in this region will provide new insights on neuron susceptibility and a better understanding of the movement-related genes/variants.

It has been observed that genes expressed in the cortex region are enriched in apoptotic processes, in response to the immune effector process [14]. Genes expressed by striatal neurons are found to be involved in neuronal projection-related processes and transport-related functions. Human-induced pluripotent stem cell (iPSC) studies have reported the genes being expressed in essential pathways such as cAMP signaling and the JAK-STAT cascade. The gene expression profile from post-mortem samples provides substantial insights into the molecular pathology of HD when compared to microarray/non-human models [15]. Innate immune signaling and its activation can be observed (in endothelial cells, microglia, and astroglial cells) at the cellular level through snRNA sequencing analysis [16]. Despite the existence of numerous mouse model studies conducted on Huntington’s disease (HD), these studies have demonstrated limited translational effects and have proven to be poor predictors of therapeutic agents and disease progression. [17]. Biomarker identification has been performed using blood samples from HD patients, while experimental validation remains challenging in these studies [18].

Previously reported studies have discussed gene expression and associated functions related to disease progression. However, these studies have not focused on the transcript-level expression profile and linked the genes with variants affecting them through GWAS mapping, QTL mapping, and transcription factor/miRNA binding. In this study, we have analyzed the expression profile at the gene/transcript level and unveiled some novel HD- associated genes by comparing them with the previously published literature. These genes are involved in neuron projection development, vascular smooth muscle cell proliferation, and are expressed in motor neurons. A deeper study of these genes will provide a clear understanding and their role in disease pathogenesis. We detected SNPs and associated them with known GWAS variants [19,20,21,22] and mapped them with reported QTL variants to understand their effect on epigenetic modifications and transcription factor binding. Further, we identified variants affecting miRNA binding and found the expression pattern of their targets in HD. In addition, essential variant genes are identified based on gene deletion studies in metabolic models. A broad literature analysis was performed to relate with the experimental evidence for the knockout of the essential variant genes identified from our analysis. We linked the expression signature with variants of HD and analyzed the functional relationship between the variant genes (VGs) and differentially expressed genes (DEGs). Enrichment studies were performed to further elucidate the role of genes identified in HD-associated disease processes. From these functional annotation studies, the majority of the variant genes and DEGs from this study are specific to immunoreactivity. Therefore, we propose that the role of upregulated aberrant neuronal inflammatory response-related genes, which may contribute to neuronal death, should be experimentally verified in terms of their reactivity in disintegrating protein aggregation. It is essential to investigate whether these genes have the potential to counteract protein aggregation and its detrimental effects.

## 2. Methods

### 2.1. Dataset, Pre-Processing, and Variant Analysis

The sequencing data of the primary motor cortex (Brodmann area 4) from the tissue of HD patients and control samples (Supplementary Table S1) were obtained from the SRA database (SRP072463) [23]. Samples are from human post-mortem tissues, and their ages range from 45to 60 and 45 to 65 for control and HD samples, respectively. Sequenced data are HD samples graded based on Vonsattel grading [24], which varies from 0 to 4 based on degeneration of the tissue. Grade 0 is neurologically enriched while grade 4 has a 95% loss of neurons. The samples collected belong to grades 2, 3, and 4. The RNA-seq data are in FASTQ format, and the data are pre-processed using the NGSQCToolkit [25], FastQ Groomer [26], Trim Galore [27], and FastQ Trimmer tools for removing low-quality reads (based on Phred score) and adapter and hexamer contamination. The detailed workflow is presented in Supplementary Figure S1. The pre-processed reads were mapped with the latest human genome (hg38) using STAR (**S**pliced **T**ranscripts **A**lignment to a **R**eference) [28], a spliced aligner for mapping reads to the reference genome. PCR duplicate removal was performed on mapped reads using Rmdup [29]. Transcriptome dataset [23] encompassing both coding and non-coding sequences is used for variant detection. Variant calling was carried out using the SAM tools—Mpileup [30] and VarScan [31]—and the variants were filtered using the stringent criteria: (1) Phred score > 30 for reflecting the reliability of base calls, (2) minimum read depth is 10 for an adequate level of coverage, (3) transition/transversion ratio is >2 for distinguishing different types of genetic changes, (4) local alignment refinement for alignment accuracy, (5) minimum allele frequency threshold is 0.01 for obtaining significant variants, (6) false discovery rate (p-adj) < 0.001 for reducing false positives, (7) strand bias evaluation (Fischer’s exact test) *p*-value < 0.01 for effectively removing false positives from strand-specific artifacts, (8) mapping quality score > 30 for accurate read alignment, (9) retained only uniquely mapped reads with high alignment scores to eliminate spurious results. Genomic location of variants is obtained by annotation using ANNOVAR [32]. To provide specificity to Huntington’s disease related variations, variants exclusively present in all HD samples and absent in controls were considered for further analysis. Variant genes were compared with differential expression profiles of HD from GEO [33], HumanMine [34] database, and Enrichr [35]. The identified variants were cross-referenced with various GWAS studies of other neurodegenerative disorders such as Alzheimer’s disease, Parkinson’s disease, and Amyotrophic Lateral Sclerosis [19,20,21,22] to establish concurrence with existing genetic associations.

### 2.2. Effect of Variants over Regulatory Mechanisms and Finding Essential Variant Genes

Variants that affect transcription factor binding were analyzed using SNP2TFBS [36]. TRRUSTv2 [37] database is used to find the associations between transcription factors and variant genes. We compared our variants with miRNASNP-v3 [38], a database of disease-related variants in miRNAs, and polymiRTS [39], a repository of variants in experimentally valid seed regions and target sites. miRNA–target interactions are retrieved from miRTarbase [40]. Gene targets from these interactions are filtered based on their expression in HD. We checked if both the miRNAs and targets are previously reported to be associated with HD. Moreover, a comparison of our variants with various XQTL studies available at QTLbase [41] was performed, to filter variants that are associated with known QTLs from other neurodegenerative diseases. We examined their involvement in multiple regulatory mechanisms such as splicing, expression, acetylation, and methylation and identified the essential variant genes based on genome-scale metabolic modeling by performing gene deletion in the Recon3D model [42], which is available at https://www.vmh.life/#home (accessed on 2 January 2023). A comparison of variant genes was performed with the metabolic genes of the model, and single-gene deletion was performed using COBRAToolBox v3.0 [43] in MATLAB. A growth rate cut-off of 990 was considered to filter the essential genes whose growth rate is reduced when compared with the growth rate of normal human metabolic model. We also noticed that the number of reactions deleted due to the respective essential gene is significantly larger than the other genes.

### 2.3. Differential Gene/Transcript Proportion Expression Analysis

Salmon [44], a transcriptome-based aligner tool, was used for finding transcript abundance with the transcriptome sequences of the human reference genome hg38. Tximport [45] was used to convert the transcript abundance to gene quantification, and DESeq2 [46] was used to identify the differentially expressed genes. Filtering of DEGs was performed based on fold change (|log_2_foldchange| > 1) and a false discovery rate (<0.05). We compared DEGs with already-reported genes of HD in the literature, HumanMine [34], and Enrichr [35]. Differential transcript expression and usage are calculated to observe transcript-level changes between two conditions (control and disease). We quantified transcript expression and proportions using DRIMSeq [47] and DTUrtle [48] to reveal transcript usage patterns in both HD and control samples.

### 2.4. Functional Enrichment and Tissue-Specific Network Interaction Analysis

Large-scale interaction network analysis is essential to elucidate the relationship between the DEGs, variant genes, and transcription factors. We constructed tissue-specific functional interaction and co-expression networks among variant genes, differentially expressed genes, and transcription factors using Reactome FI [49], HumanBase [50], and coExpressDB [51]. Additionally, the construction of a gene–gene interaction network was performed using the STRING database exclusive for the differentially expressed genes from our study, and they were clustered based on their function. To understand the enriched pathways and molecular functions, we performed functional enrichment using ClueGO [52], clusterProfiler [53], KEGG [54], and InterPro [55] databases.

## 3. Results

### 3.1. Differential Gene/Transcript Expression Profile

**Figure 2:**
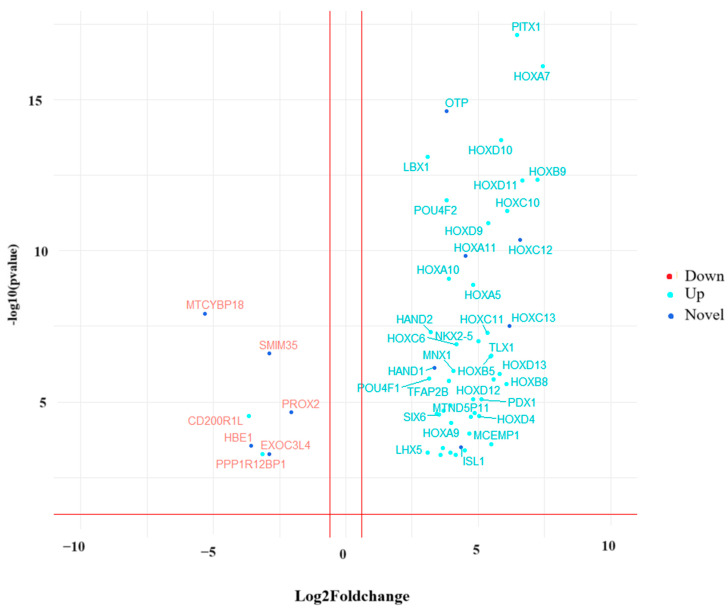
Volcano plot of differentially expressed genes. Genes are filtered based on fold change (|log_2_foldchange| > 1).

We performed transcriptome-based quantification using salmon and found 55 differentially expressed genes; among them, 27 are already reported in HD, and 28 are novel genes identified in this study. A volcano plot of differentially expressed genes is shown in Figure 2.

A total of 22 *HOX* genes are differentially expressed in BA4 samples, as already discussed in the literature [56]. Although the importance of *HOX* genes in HD has previously been studied [57], their role in disease is yet to be understood deeply. We found that the majority of genes are indulged in neuroinflammation/neurodegeneration, and their upregulation is observed in the disease samples. Hence, they can act as potential candidates for gene knockout studies for HD. *FOXL2* is a downregulated gene and is responsible for apoptosis/regulation of cell death/survival, as previously reported [58]. Both *CTAG2* and *FOXL2* are predicted to be involved in regulating the MAPK cascade and GPCR signaling. *CD200R1L* and *EXOC3L4* are found to be differentially expressed in microglia-enriched genes and astrocyte-enriched genes, respectively [59]. The function of novel genes such as *HBE1* and *SMIM35* has to be understood to elucidate their role in HD pathogenesis. The novel gene *MTCYBP18* [60] has been identified to play a role in the transport of cytochrome B during metabolic processes. Interestingly, in Huntington’s disease (HD), this gene is found to be downregulated. This downregulation suggests that disturbances in the metabolic process can impact the neuronal survival pathway and potentially disrupt the homeostasis of brain function. It highlights the potential significance of *MTCYBP18* in understanding the mechanisms underlying HD and the importance of metabolic processes in maintaining optimal neuronal function and survival [61]. Novel genes identified in this study are majorly involved in Wnt signaling, MAPK cascade, and regulation of cell differentiation processes. The expression signature of some of the novel genes is discussed briefly in the section below.

#### 3.1.1. Novel Gene Expression Pattern in BA4

*HOXA11* is involved in the negative regulation of cell proliferation and is also a member of the Wnt signaling pathway. Additionally, it is involved in the GPCR signaling pathway and positively regulates the MAPK cascade. These signaling pathways are positive controllers of neuroinflammation [62,63,64,65], and their members are contributors to neuron degeneration. Its anti-sense RNA (*HOXA11-AS*) is responsible for neuroinflammation through microglia [66]. *MNX1* is a novel HD (BA4) gene responsible for neuron projection development, neurogenesis, neuron differentiation, and nervous system development. It has previously been observed that *MNX1* is expressed in zebrafish motor neurons [67]. Its activity can be further studied in the HD brain to understand its role in disease pathogenesis. *MMP9* is an important participant in the regulation of vascular smooth muscle cell proliferation [68], which is a crucial process leading to neurodegeneration in HD [69]. *CCL3L1* is a chemokine gene that actively involves cytokine/interleukin signaling, which is responsible for inflammatory activities [70] in HD.

**Figure 3:**
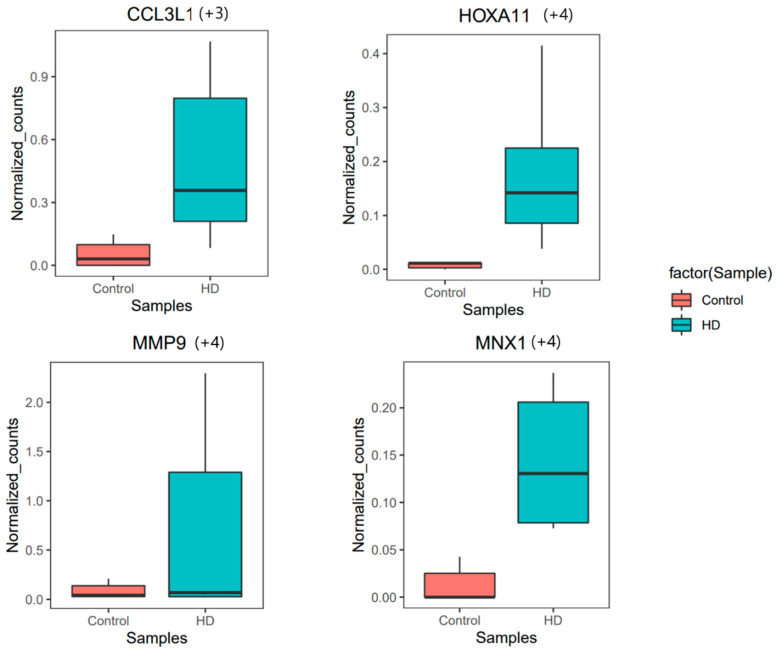
Box plot showing upregulated genes in HD. The box and the line inside the box represent the interquartile interval of the data (normalized counts) and median, respectively. The whiskers connecting the box on both sides towards the top and bottom are the maximum and minimum values in the data, respectively. The adjusted *p*-value (p_adj_) is less than 0.05; the + sign denotes upregulation. The numbers in parentheses represent the fold change.

These genes discussed above are upregulated in our study, and the transcript expression plots for the genes are shown in Figure 3. A deeper study was also carried out to understand the expression of each gene through transcript expression/usage analysis. In summary, these novel genes exhibit dysregulation in both gene and transcript expression patterns. These genes are known to be involved in the Wnt signaling pathway, and the dysregulation of this pathway may have implications for brain vascular function [71,72]. Understanding the impact of these dysregulated genes on the Wnt signaling pathway and neuroinflammation could provide valuable insights into the underlying mechanisms that influence brain vascular function and neurodegeneration in HD.

#### 3.1.2. Differential Transcript Expression/Usage Analysis

**Figure 4:**
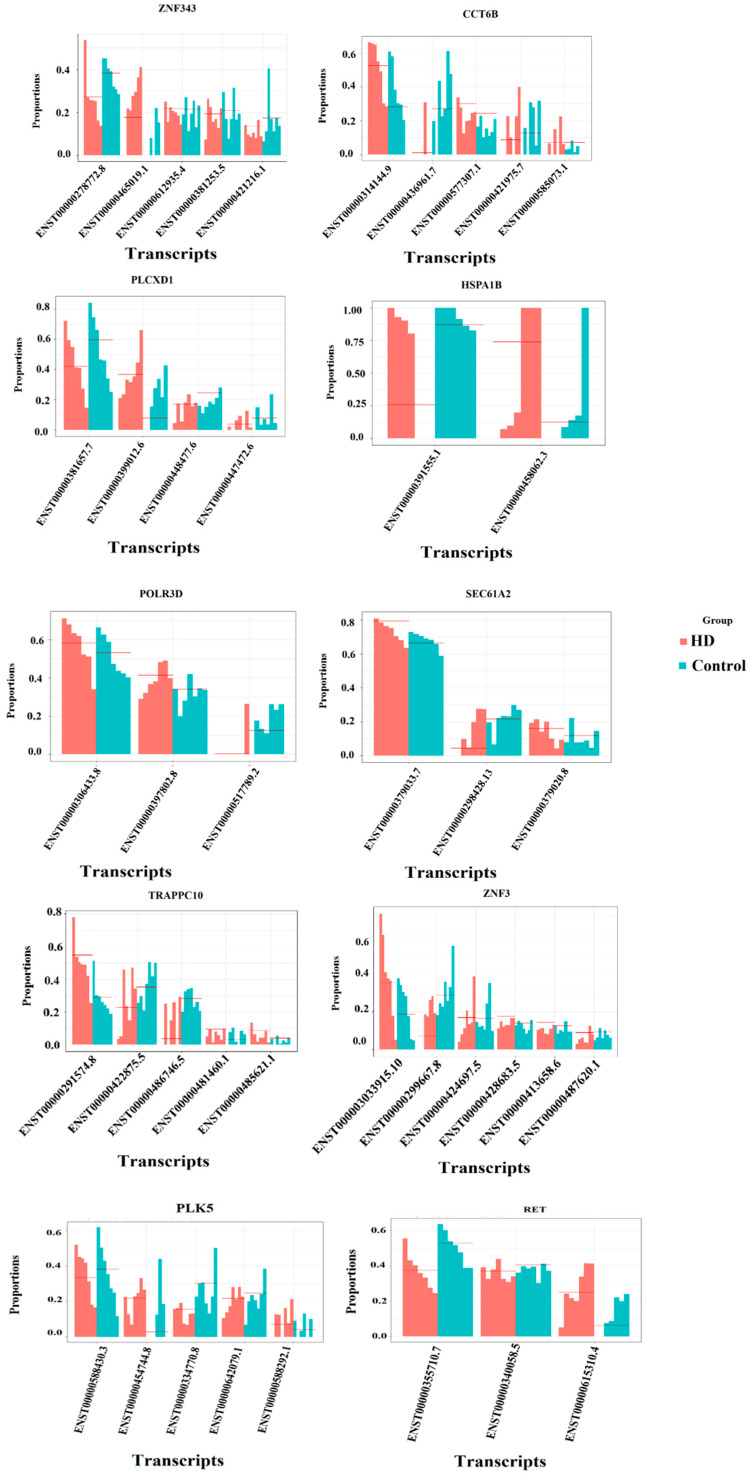
Transcript proportion plot for differentially expressed genes. The x-axis represents the transcripts of a gene, and the y-axis represents the proportion level (isoform relative abundance between HD and control).

Differential transcript expression is a transcript-level expression in which expression change is observed in any one of the transcripts of a gene between the control and HD. Transcript usage analysis is performed to identify the contribution of individual transcripts (transcript/isoform composition of the gene) to the expression of genes between HD and control samples. We obtained 175 genes with 241 significant transcripts and observed the proportion difference between different transcripts, which are detailed in Supplementary Table S2. Genes that are differentially expressed at the isoform level are found to be playing an important role in the inflammatory response. The transcript proportion plots for the genes involved in the inflammatory response and neuron differentiation process are given in Figure 4.

*RET* plays a crucial role in the positive regulation of neurogenesis [73] and neuronal differentiation. It is responsible for neurodevelopment and is involved in key functions such as stress response and motor function. Notably, alterations in *RET* have been implicated in Parkinson’s disease, suggesting its significance in the pathogenesis of this neurodegenerative disorder. *PLK5* is a crucial gene involved in learning and memory expressed mainly in the hippocampus; this gene’s function is altered in Alzheimer’s disease [74]. These genes, *RET* and *PLK5*, play a significant role in movement-related functions, but their expression is downregulated in Huntington’s disease (HD). The under-expression of these genes may contribute to the motor dysfunction observed in HD patients. Further research is needed to fully understand the mechanisms underlying the downregulation of *RET* and *PLK5* in HD and their specific impact on movement-related functions.

#### 3.1.3. Similarity of Gene Expression with Previous Bulk RNA-Seq and GTEX Datasets

**Figure 5:**
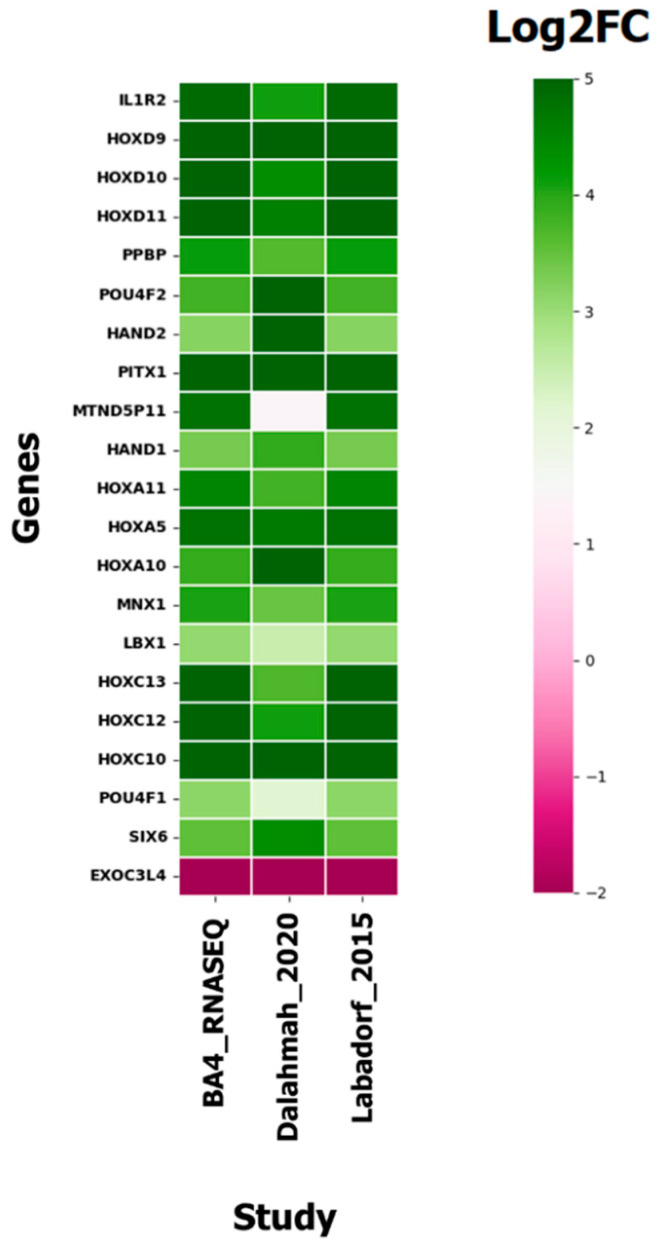
Comparison of genes in HD BA4 RNA-seq with reported bulk RNA-seq expression data using heatmap (Log2FC is the fold change value of genes in each study).

The comparison of differentially expressed genes of BA4 samples was carried out with previously reported [57,75] RNA-seq (bulk and single nucleus RNA-seq) studies to validate the differential gene expression pattern. Since the expression results from the BA4 region are not available in multiple studies, we compared our DEGs with genes reported in other cortex tissues (prefrontal cortex (BA9), cingulate cortex). It is important to know if the differentially expressed genes in HD are exhibiting similar expression as per previously reported studies. We observed similar expression patterns when compared with other RNA-seq studies of different brain regions, and the results are shown in Figure 5.

**Figure 6:**
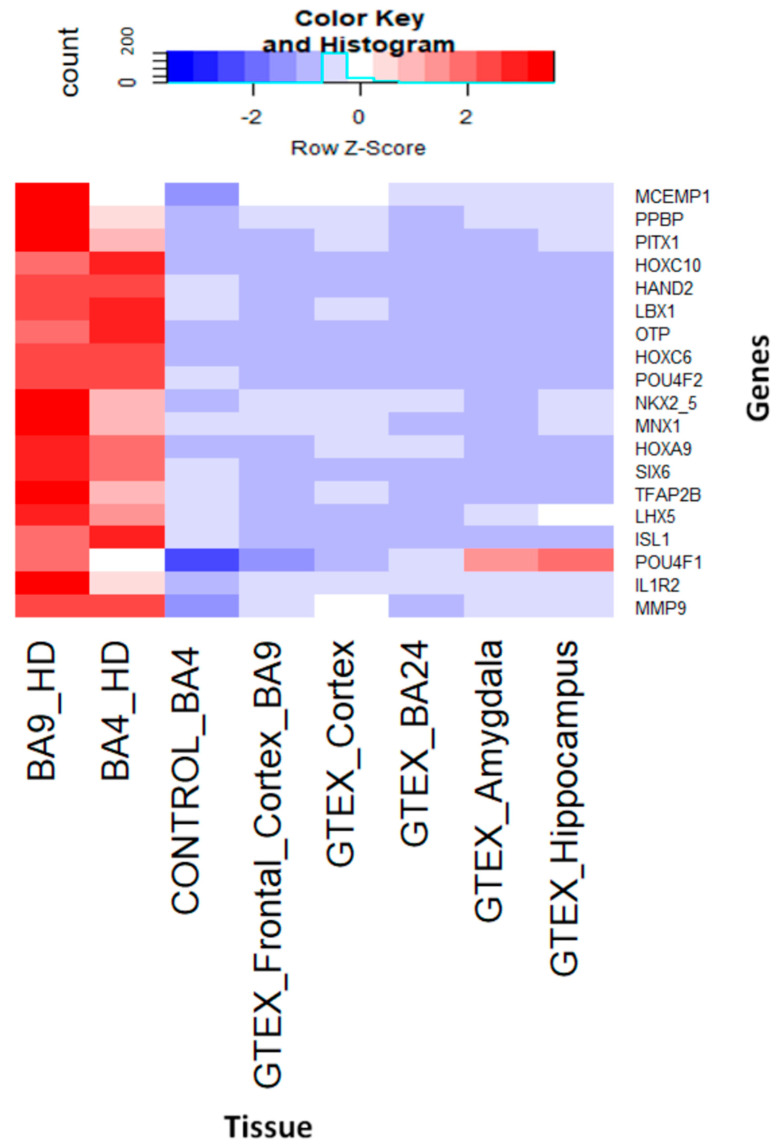
Comparison of the gene expression profile of BA4 with GTEX tissues of the brain.

We conducted a comparison of the gene expression profile between HD BA4 (Huntington’s disease in Brodmann area 4) samples and normal (non-disease) tissue samples obtained from the GTEX portal (Figure 6). This analysis revealed distinct differences in the gene expression patterns of BA4 affected by the disease compared to control samples. To illustrate this comparison, Figure 5 depicts the contrasting gene expression profiles of both novel and reported genes specific to Huntington’s disease BA4, juxtaposed with GTEX tissue samples.

### 3.2. Variant Analysis and Associating Variant Effect on Disease-Associated Regulatory Mechanisms

Variants play a crucial role in different regulatory mechanisms directly or indirectly through histone modifications, changes in gene expression, and transcription factor binding that may regulate the gene expression pattern. The study of the effect of variants is important in understanding their role, for finding therapeutic targets, and in drug repurposing [76]. By comprehending the effects of variants and extrapolating their roles in various regulatory mechanisms, we can gain a comprehensive understanding of each variant’s contribution to the regulatory landscape.

The identified variants are found to be majorly positioned in non-coding regions of the genome. These variants are compared with the already-reported genotype molecular trait associations to interpret their role in epigenetic modifications such as splicing, expression, and histone acetylation/methylation. Our results showed that most of the variants have a huge role in regulating expression. Out of the 20,917 variants, 11,446 of them are involved in expression regulation (eQTL). In total, 3464 and 1104 variants are actively involved in regulating splicing and alternative polyadenylation, respectively (Supplementary Figure S2). The least number of variants is involved in regulating RNA editing and methylation processes. We hypothesize that variants of HD have a prominent role in regulating gene expression. These variants play a critical role in the pathogenesis of HD by actively modulating the gene expression. We have verified the reliability of our predicted variants by comparing them with disease-associated variants of different GWAS studies performed earlier [19,20,21,22].

#### 3.2.1. Role of Variants on Transcription Factor Binding

**Figure 7:**
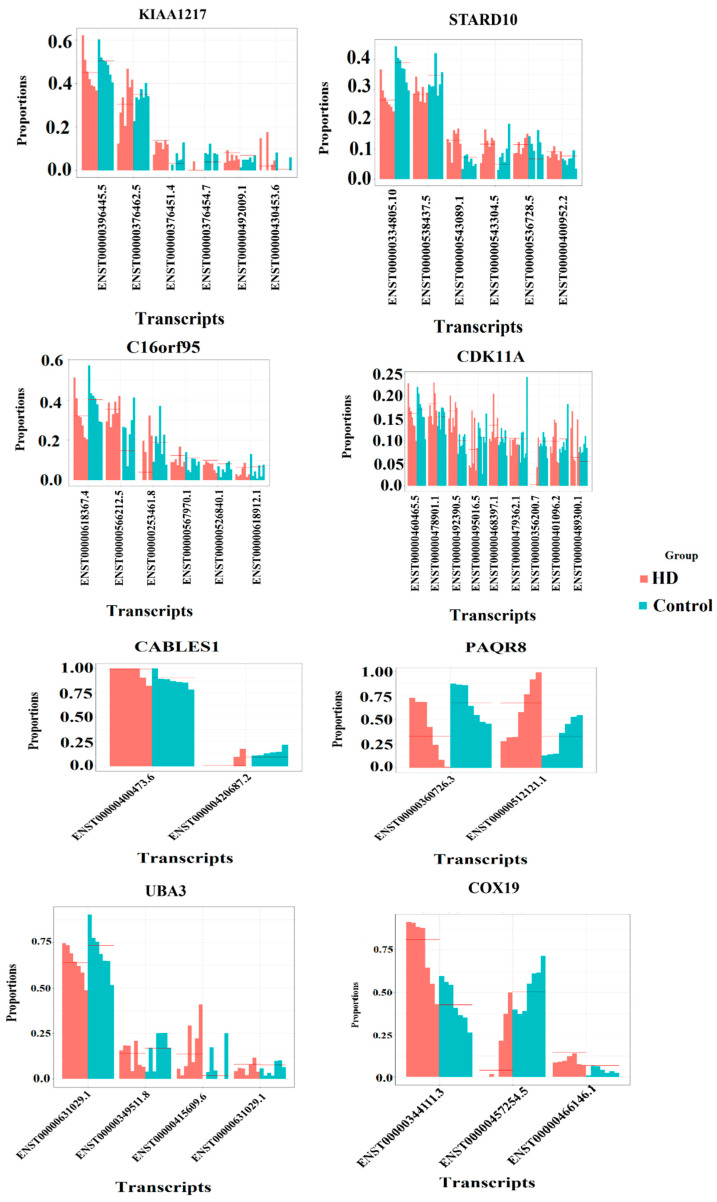
Transcript proportion plot of variant genes that are altering transcription factor binding. The x-axis represents the transcripts of the gene, and the y-axis represents the proportion level (isoform relative abundance between HD and control).

Most of the disease-associated variants are responsible for altering transcription factor binding and accelerating disease progression. Variants identified are observed to overlap with transcript position (ensemble overlapped transcript position of variants), and these transcripts are also found to be differentially expressed in HD through DTE/DTU analysis. In Table 1, we present the variant information that is altering the binding of transcription factors, which are dysregulated in HD. It also has information on differential transcripts overlapping with the variant. The proportion plots of all the variant-associated genes are shown in Table 1 in Figure 7.

*COX19* is a gene involved in the assembly and function of cytochrome c oxidase (COX), which is a crucial enzyme in the mitochondrial respiratory chain. In the brain, energy production is essential for neuronal function and synaptic activity. The *COX19* variant gene affects the binding of transcription factor *ADAP1*, and it is responsible for regulating cell ion homeostasis [77]. The variant associated with *COX19* is rs10282027, which overlaps with transcript ENST00000457254.5 and is differentially expressed among HD samples. The affected transcription factor (*ADAP1*) is found to be downregulated in HD, and it is enriched in regulating GTPase activity [78]. The neuroprotective effect is the role of GTPase activity in HD [79]. Hence, the variant effect on transcription factor binding indirectly entombs the neuroprotective effect of the gene. The proportion plot for the gene *COX19* is shown in Figure 7.

*UBA3* is a gene encoding a protein called ubiquitin-like modifier-activating enzyme 3. Ubiquitin is involved in protein degradation, regulation of protein activity, and cellular signaling. In the brain, *UBA3* may participate in processes such as synaptic plasticity, neuronal development, and neurotransmitter release, thereby influencing brain functionality [80,81]. We identified that *EOGT* is a crucial transcription factor that is downregulated and affected by the variant rs3853156 (*UBA3*). It is also observed that *EOGT* is an important gene to regulate glycosylation. Irregular glycosylation leads to abnormal neuronal functions in HD [82]. The variant is overlapped with the transcript (ENST00000415609.6), which is differentially expressed among HD when compared with control samples. Variant gene *UBA3* is an important regulator of immune activity [83], and its proportion plot is shown in Figure 7. We propose that variant genes that are immune regulators can alter the binding of transcription factors, which causes irregular glycosylation in HD.

*CDK11A* has been associated with neuronal migration, neurite outgrowth, and synapse formation [84]. It may contribute to the proper development and maintenance of brain circuits. *CDK11A* is a variant gene that is responsible for growth-related functions [85] and alters the binding of three transcription factors, *GNB1*, *PAX5*, and *ZFX*, which are downregulated and participants of crucial pathways such as GPCR signaling, MAPK cascade, and Wnt signaling. These signaling pathways have a neuroprotective role in HD patients [86,87]. *PAQR8* [88] is a variant gene that is responsible for functions such as inhibition of apoptosis and neurite outgrowth and is observed to be affecting a downregulated transcription factor *IRF1*, a key immune regulator. The variant gene *KIAA1217* is responsible for the regulation of growth-related functions and disrupts the binding of an upregulated transcription factor *SOX5*, an essential gene in the neurogenesis process.

In summary, variant genes play a significant role in modulating the expression and regulatory effects of transcript expression patterns, as well as influencing transcription factor binding indirectly. In Huntington’s disease (HD), the genes responsible for maintaining homeostasis and promoting growth are downregulated. In our study, we have demonstrated an association between regulatory changes associated with variants and changes in transcript expression, providing valuable insights into the profound role of disease-associated variants. These findings contribute to a deeper understanding of the impact of such variants on disease pathology.

#### 3.2.2. Role of Variants on miRNA Binding and Their Targets

**Figure 8:**
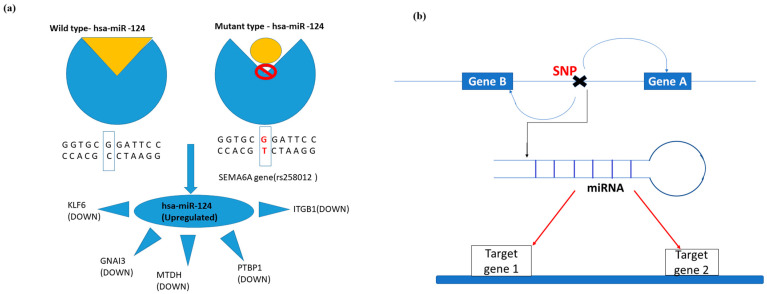
Representation of the effect of variants on miRNA binding and their targets. (**a**) The miRNA (has-miR-124) binding alteration by variant rs258012 and the downstream genes and expression of miR-124. (**b**) An illustration of genes associated with miRNA before and after binding.

Variants affecting the binding of miRNA are potential candidates in neurodegeneration. There is evidence that variants located in seed regions of miRNAs have a deleterious effect on gene expression and its role is also reported in different mechanisms favoring neurodegeneration [89]. A variant that alters the binding of a miRNA can indirectly regulate the expression of the miRNA gene targets. We predicted variants affecting the binding of miRNA by comparing with repositories, polymiRTs, and miRNASNP-v3 that have variants in experimentally verified seed regions and target sites. Predicted variants and their corresponding miRNAs are compared with previously reported studies in HD for their expression. miRTarbase is used to find the gene targets of miRNAs, and their targets were compared with differentially expressed genes. The variant rs258012 and its associated gene *SEMA6A* disrupt the binding of hsa-miR-124, as shown in Figure 8.

Hsa-miR-124 is an upregulated miRNA [90] in HD, and it targets five differentially expressed genes in HD. *ITGB1* is a downregulated gene that performs critical functions in HD such as regulation of angiogenesis [91], vasculature development, and protein kinase signaling [92]. These are critical functions in HD, and the dysregulation of *ITGB1* can have an adverse effect on HD patients. *GNAI3* is also a downregulated gene that is a target of miR-124 and is a participant in GPCR signaling [93], blood morphogenesis, dopamine receptor signaling pathway, and regulation of autophagy [94]. The under-expression of *GNAI3* due to miR-124 can have derived consequences on neurodegeneration due to autophagy and protein aggregate deposits. *MTDH* involves the regulation of angiogenesis [95], NF-KappaB signaling [96], and autophagy [97], which is downregulated in HD and a gene target of miR-124. Silencing the expression of mir-24 can have a positive impact on HD pathogenesis. miR-124 targets *PTBP1* (downregulated in HD) and can impact disease-causing mechanisms such as muscle coordination and alternative splicing effects. *PTBP1* is an active member in regulating alternative splicing events [98] and muscle cell differentiation [99]. *KLF6* is a downregulated gene targeted by miR-124 and is responsible for regulating NF-KappaB transcription factor activity and the ROS biosynthetic process [50]. In summary, the importance of miR-124 in HD and its disease progression can be unfolded by analyzing the variant/variant-associated genes and investigating its targets to obtain potential therapeutic agents. The effect of variant genes on miRNA binding and their effect on the miRNA target genes is shown in Table 2.

The variant-associated genes *HOXA10* and *HOXD9* affect the binding of miRNA-29-3p, which is upregulated in HD. Mir-29-3p targets four downregulated genes in HD (*COL4A2*, *KLF4*, *ITGB1*, and *COL1A2*) that are involved in vasculature development. Brain vasculature is important in HD [100]. *COL4A2* encodes a component of type IV collagen, which is a major constituent of the extracellular matrix in the brain’s blood vessels. It plays a critical role in maintaining the structural integrity of the blood–brain barrier and the proper functioning of brain vasculature [101]. *KLF4* is a transcription factor that regulates the expression of various genes involved in cell proliferation, differentiation, and development [102,103]. In the brain, *KLF4* is important for neuronal survival, synaptic plasticity, and neuroinflammatory responses. *ITGB1* encodes an integrin protein involved in cell adhesion and signaling [104]. It plays a crucial role in mediating interactions between neurons and the extracellular matrix, influencing processes such as neuronal migration, axon guidance, and synaptic connectivity. *COL1A2* encodes a component of type I collagen, a key structural protein in the brain’s extracellular matrix. It provides mechanical support to brain tissue and contributes to organization and stability [105]. Downregulation of these genes may affect homeostasis integrity and affects brain function. We also found variants associated with the gene *KLC1*, which affects the binding of miR-34a-5p that is upregulated in HD and targets *ERBB2*, *SRC*, *GAS1*, and *PDGFR* (downregulated genes). These genes contribute to various aspects of brain function, including neuronal development, synaptic plasticity, cell growth and survival, and maintenance of brain cell populations [106]. Similarly, we identified the variant-associated genes that affect the miRNA which is downregulated (miR-146a-5p), and their associated target genes (*SOX2*, *ERBB4*, *BCLAF1*, and *RAC1*) are upregulated in HD. These target genes collectively contribute to the intricate regulation of the MAPK pathway in the brain, impacting important cellular processes such as cell fate determination, synaptic development, gene expression, cell survival, and cytoskeletal dynamics [50].

In summary, our findings demonstrate that variant genes have an impact on miRNA binding, which in turn indirectly affects dysregulated downstream genes in Huntington’s disease (HD). These target genes play essential roles in crucial cellular functions, including the MAPK cell survival pathway and blood–brain barrier functionality. Detailed information on variants affecting miRNAs is shown in Supplementary Table S3.

#### 3.2.3. Essential Gene Identification from Genome-Scale Metabolic Model of Variant Genes

Essential variant-associated genes are identified from genome-scale metabolic model analysis (Supplementary Figure S3). We have considered the human metabolic model, Recon3D [42], for finding essential variant-associated genes through single-gene deletion. Recon3D contains 3695 metabolic genes and 13,543 reactions associated with them. The comparison of variant genes with metabolic genes of Recon3D was carried out, and common downregulated genes were selected for the single-gene deletion process. Single-gene deletion of the 839 variant genes (downregulated) was performed, and the observed growth rate changes and affected reactions due to the deletion were identified. The recon3D model contains metabolic genes and reactions, and the model has an objective function, which denotes the growth requirements of the cell. The growth rate of the human metabolic model is 1000. A total of 65 genes had a reduction in growth rate when in silico knockout was performed. Growth rate ratio (grRatio) is computed using a knockout study, and essential genes are screened using grRatio, similar to the study performed previously [107]. grRatio is a ratio between the growth rate of the knockout model and the growth rate of the wild-type model. A growth rate ratio greater than 0.9 is considered for filtering essential genes. A systemic review of the literature was performed to find out the importance of these essential genes and the impact of their deletion on various mechanisms/functions in HD. Thirty-two genes are predicted as essential genes based on the grRatio filter and experimental evidence of knockout studies. The details of essential variant genes are given in Supplementary Table S4 ([108,109,110,111,112,113,114,115,116,117,118,119,120,121,122,123,124,125,126,127,128,129,130,131,132,133,134,135,136,137,138,139,140]). Most of these genes are part of metabolic processes and energy demands of the brain. Downregulation of these essential genes can impact the metabolic pathways and cause stress within mitochondria.

### 3.3. Function Interaction Network of Differentially Expressed Genes and Transcription Factors

**Figure 9:**
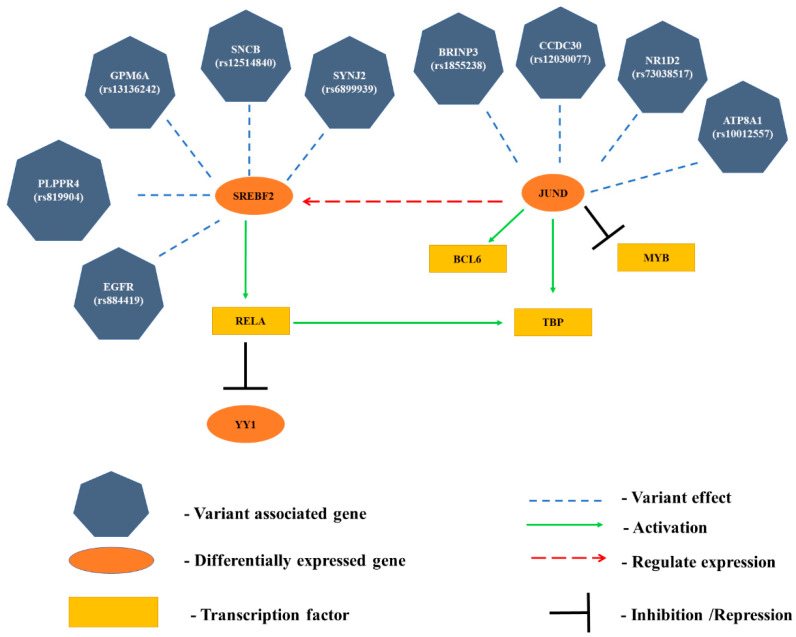
Function interaction network between variant-associated genes, differentially expressed genes, and transcription factors.

We constructed a functional interaction network between our differentially expressed genes, variant-associated genes, and the transcription factors whose binding is affected, which is shown in Supplementary Figure S4. We used Haploreg, TRRUST [4], and HIPPIE for deriving the functional relationship between the genes and transcription factors. Based on the network parameters (Supplementary Figure S4, the grouping of hub genes is performed based on degree and betweenness centrality. Some of the hub genes and their interactions are illustrated in Figure 9. *SREBF2* is an upregulated gene that is activated by five variant-associated genes. *HOXA5* also activates *RELA* (upregulated in HD). *SREBF2* is a crucial participant in aggregation and nutrient deprivation [141], which in turn activates *RELA*, a member of the T-cell receptor signaling pathway, and regulates the apoptotic signaling pathway [142,143]. *RELA* is a transcription factor that inhibits (transcriptionally represses) the HD gene *YY1*. *YY1* is a transcription factor in HD, which is an important member regulating the production of interferon β [144] and DNA repair [145]. *RELA* also activates *TBP*, an upregulated gene of HD. A cascade of activations can be visualized in Figure 9, where variant-associated genes are activating *SREBF2*, which activates the transcription factor *RELA*. The sequence of activations can contribute to major pathways leading to neurodegeneration in HD. It also shows the activation of *JUND*, an upregulated gene of HD by the four variant genes *BRINP3*, *CCDC30, NR1D2*, and *ATP8A1*. *JUND* is a well-known gene for its involvement in ESR signaling [146] and anti-apoptotic activities. *JUND* is an upregulated HD transcription factor that regulates the expression of *SREBF2* and activates two HD genes: *BCL6* and *TBP*. *BCL6* and *TBP* participate in functions that include the regulation of cell growth [147] and the transcription initiation process [148], respectively. *JUND* also inhibits/blocks the expression of transcription factor *MYB*. *MYB* is a crucial gene in activating the immune response in cells [149]. Important genes such as *YY1* and *MYB* are inhibited by other upregulated genes which can lead to loss of function or inactivation of genes that have a neuroprotective effect [150]. To summarize, we have constructed the function interaction network based on the regulatory associations between differentially expressed genes and transcription factors obtained from the Reactome knowledgebase. We have illustrated some interactions among hub genes in Supplementary Table S5.

### 3.4. Differential Co-Expression Network of Genes and Function Enrichment Study

**Figure 10:**
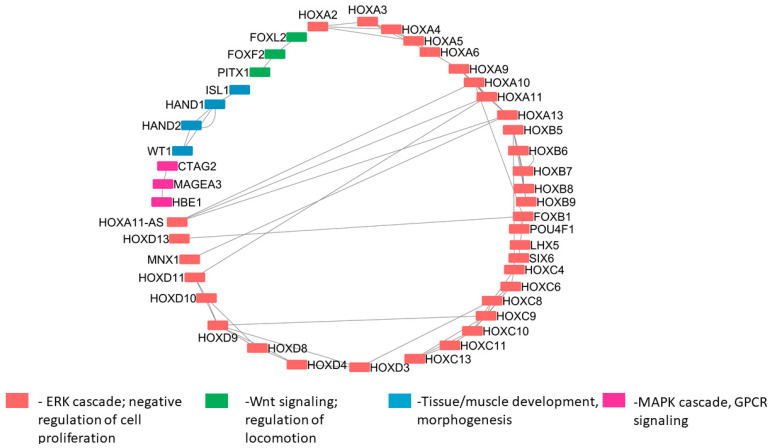
Co-expression network of differentially expressed genes.

We constructed a co-expression network for differentially expressed genes and clustered them based on their role in different biological processes. It is observed that co-expressed genes involved in similar pathways stay connected in the network. For example, in Figure 10, green color nodes are involved in locomotion-related functions and Wnt signaling. Similarly, blue color nodes include *ISL1*, a novel gene co-expressed together with *HAND1* and *HAND2*. These genes play a key role in tissue/muscle development [151], which is essential in HD.

**Figure 11:**
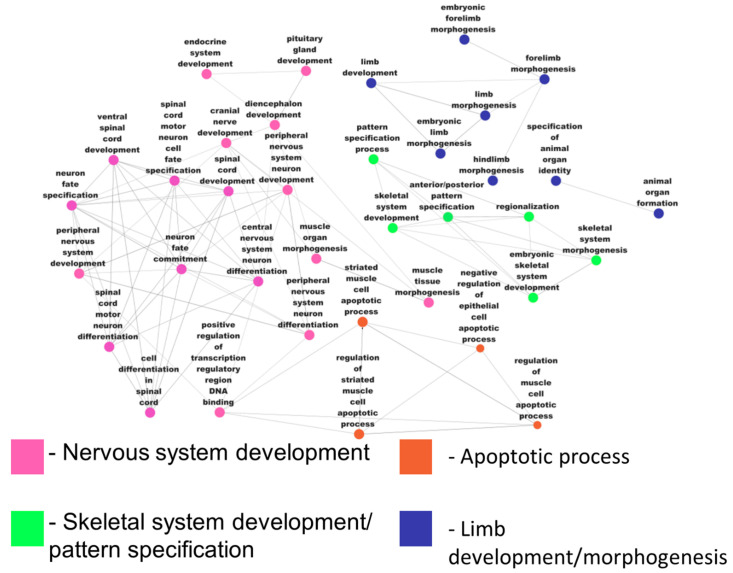
Tissue-specific functional module network of differentially expressed genes (functionally grouped annotation network of DEGs).

Functional module enrichment analysis was performed on differentially expressed genes, and the enriched genes were categorized based on the function in which they involve. Most of the DEGs are observed to be involved in neuronal differentiation. Armstrong et al. [152] have observed neuronal differentiation in HD and discussed its importance. Most of the upregulated genes are involved in the GPCR signaling pathway, MAPK cascade, and histone modifications. Further investigations on these genes in histone modifications [153] are affecting memory or any other epigenetically regulated alterations in HD patients. Upregulated genes can be further studied for identifying potential therapeutic agents using GPCR-related targets for HD [154]. Figure 11 shows different biological processes in which the differentially expressed genes are enriched. The majority of the upregulated genes are involved in limb/organ morphogenesis and spinal-cord-related neuron differentiation. This indicates that genes of the BA4 region are crucial for motor-related symptoms, and they must be investigated for elucidating their role in disease pathogenesis.

We also examined the pathways and biological processes in which variant genes and differentially expressed genes together participate in using HumanBase. It was found that differentially expressed genes and variant-associated genes are majorly participating in vesicle transport. It is known that vesicle trafficking is one of the critical processes involved in neurodegeneration [155]. The genes that are associated with vesicle-mediated transport require further studies for understanding their role in the activation of the immune response or inflammation in the HD brain. Most of the genes are observed to be involved in neuron-related biological processes (Supplementary Figure S5). For example, *ISL1*, *HOXD10*, and *LBX1* (upregulated genes) are important regulators of many crucial molecular functions such as spinal cord development and neuron projection morphogenesis.

### 3.5. Proposed Mechanism in HD Pathology

The comparison of our variant-associated genes and differentially expressed genes to known markers concerning cell type revealed that most of the variant-associated genes are under immune response and blood–brain barrier functionality. From this, we propose that neuroinflammation involves the activation of immune cells in the central nervous system, particularly microglia and astrocytes, in response to various pathological stimuli. In the context of α-synuclein pathology and motor neuron death, neuroinflammatory signals play a significant role. Upon activation, microglia and astrocytes release pro-inflammatory cytokines, chemokines, and reactive oxygen species. Neuroinflammation can have both beneficial and detrimental effects. On one hand, the release of inflammatory molecules by these cells aims to remove harmful substances and promote tissue repair. On the other hand, excessive or chronic neuroinflammation can lead to detrimental effects, including motor neuron death. They can disrupt the balance of neurotransmitters, impair neuronal function, and promote the production of toxic molecules. Additionally, chronic inflammation can lead to the activation of immune cells and the perpetuation of the inflammatory response, further exacerbating motor neuron death.

**Figure 12:**
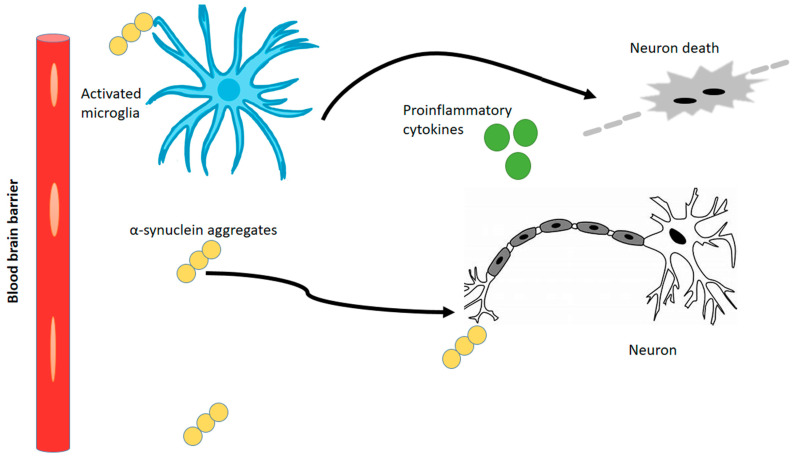
Proposed mechanism underlying neuroinflammation and neuronal cell death.

Overall, the neuroinflammatory signals originating from microglia and astrocytes in response to α-synuclein pathology contribute to the complex mechanisms underlying motor neuron death [156]. These aggregates indirectly affect disease progression by activating microglia and astrocytes through neuroinflammation, as shown in Figure 12. Understanding and modulating neuroinflammation are important areas of research for developing therapeutic strategies to intervene in neurodegenerative diseases. Studies are performed on Huntington’s disease and other neurodegenerative diseases [157,158,159,160] to implement modulation of neuroinflammation in disease models. Analyzing the upregulated variant genes of reactive glial celltypes - will give additional insights into their role in HD patients.

### 3.6. Limitations

A major limitation of this study is the small size of the data obtained from BA4 (obtained from patients and controls). Although we have compared the quantified HD expression with GTEX and other bulk RNA-seq data, we still need a large population and cell-level information (single-cell data) to understand the detailed expression patterns and variant effects.

## 4. Discussion

In the context of Huntington’s disease (HD) research, the examination of gene expression and its intricate regulatory mechanisms holds profound significance. Building upon the foundation laid by previous investigations, particularly the work of Lin et al. [23] that highlighted differential alternative splicing changes in the frontal cortex, our study delved comprehensively into the interplay among differentially expressed genes, transcripts, and variants, as well as unraveling their effects on key regulatory factors such as transcription factor binding, miRNA binding, and epigenetic modifications. This multifaceted exploration offers valuable insights into the molecular underpinnings of HD.

One of the key findings of our study involves the identification of differentially expressed genes within the BA4 region, revealing their pivotal roles in processes related to neuroinflammation and neurodegeneration. Our approach provides a broader perspective by considering not only the differential expression of genes but also the nuanced differences in transcript isoforms. Quantifying transcript-level abundances contributes to more refined differential gene expression results, an aspect that has been less explored in prior BA4 studies. Importantly, our study extends beyond mere identification to the analysis of the functional consequences of genetic variants. The genes associated with inflammatory responses are recognized as influential modulators of neurodegeneration [161], and we systematically examined their expression proportions in relation to inflammatory responses and neuron differentiation. By validating the disease associations of identified variants through comparison with large-scale GWAS data, we ensured the robustness of our findings. Through computational tools, we predicted the impact of variants on diverse regulatory mechanisms, including the alteration of transcription factor binding, which orchestrates the activation or repression of pivotal genes in the context of HD.

Our analysis provides novel insights into the modulation of transcription factor binding by variants, shedding light on a previously unexplored aspect. Furthermore, our study highlights the regulatory effects of variants on miRNA binding, utilizing experimentally verified SNPs that influence miRNAs. The incorporation of miRNA target information from the existing literature allows us to infer downstream effects on miRNA target genes, revealing an additional layer of regulatory complexity. An integral component of our study involves exploring the interplay between genetic variants and differentially expressed genes and transcripts. This nuanced analysis reveals the overlap between variant-associated genes and differentially expressed transcripts within the study. Importantly, this investigation is a unique contribution to the dataset analyzed by Lin et al., as variants and associated genes were not previously scrutinized in BA4 RNA-seq samples.

A distinctive facet of our study is the exploration of the impact of variants on post-translational modifications, a dimension rarely explored in the context of the BA4 region. In addition, we predicted essential metabolic genes that have a significant bearing on enzymatic and metabolism-based functions within the HD brain. This prediction was rigorously validated through extensive literature analysis, establishing links to supportive evidence from gene knockout studies. The correlation between neurodegeneration and metabolic changes within the brain is a well-established paradigm [162]. Our findings hold promise in addressing energy imbalances in HD patients by identifying and studying essential metabolic genes that could potentially mitigate the associated energy demands. To unravel the intricate interactions, we constructed functional interaction networks and co-expression networks. Enrichment analysis provided valuable insights into the specific pathways and biological functions in which the identified genes actively engage, contributing to the overall progression of the disease. This holistic perspective further strengthens our understanding of the complex mechanisms underlying Huntington’s disease.

In summary, our study extends the boundaries of previous research by comprehensively exploring the landscape of gene expression, transcript isoforms, and variant interactions within the context of Huntington’s disease. By investigating the multifaceted regulatory mechanisms at play, we have contributed to a deeper understanding of the molecular intricacies that govern disease progression, potentially paving the way for targeted therapeutic interventions.

## 5. Conclusions

In our study, we investigated the transcriptomic patterns in HD patient samples to gain insights into differentially expressed genes and transcripts. We observed that upregulated genes primarily contribute to neuroinflammation and neuron death processes, while downregulated genes play important roles in crucial pathways such as MAPK and signaling pathways. Through variant analysis and effect prediction, we discovered that these variants have disease-associated regulatory roles. They affect transcription factor binding and miRNA binding, and their overlapped transcripts are associated with differentially expressed transcripts. We identified novel variant genes such as COX19 and CDK11A, which are enriched in neuroprotective functions and alter the binding of downregulated transcription factors in HD. Furthermore, we observed variants that impact the miRNA binding of upregulated miRNAs such as miR-29-3p, miR-34a-5p, miR-124, and miR-196a-5p. The gene targets of these miRNAs are downregulated and participate in pathways such as the ERK cascade and vasculature development. Differential gene expression analysis highlighted the significant involvement of upregulated genes in neuroinflammation and neuron degeneration. To understand the relationship between novel differentially expressed genes and variant genes, we constructed BA4-specific interaction networks. Through our comprehensive multi-omic analysis, we propose that genes involved in neuroinflammatory signaling have a detrimental impact on neuronal survival and significantly affect motor neuronal functionality. These genes can disrupt vascular integrity, leading to compromised blood flow and nutrient supply to the brain. Additionally, they can affect energy metabolism processes, further impairing the proper functioning of motor neurons. The dysregulation of these genes highlights their critical role in the pathological mechanisms underlying neurodegenerative diseases and emphasizes the importance of targeting neuroinflammatory pathways for therapeutic interventions aimed at preserving neuronal health and motor function.

## Figures and Tables

**Table 1:** Variants’ effect on transcription factor binding/transcript proportions.

Chr	REF/ALT	Gene (Expression)	Variant	Transcripts	Variant Novel/Reported	QTL Category	Transcription Factor Affected	Transcription Factor Expression
Chr7	G/A	*COX19* (UP)	rs10282027	ENST00000457254.5	Novel	eQTL	*ADAP1*	DOWN
Chr3	T/C	*UBA3* (UP)	rs3853156	ENST00000415609.6	AD, PD, AMD	eQTL	*EOGT*	DOWN
Chr1	T/C	*CDK11A* (DOWN)	rs1137005	ENST00000356200.7	NOVEL	eQTL, sQTL	*GNB1*	DOWN
Chr6	T/A	*PAQR8* (DOWN)	rs78305768	ENST00000512121.1	AD, AMD	eQTL	*IRF1*	DOWN
Chr16	C/T	*C16orf95* (UP)	rs12148919	ENST00000562840.1	AD, AMD	eQTL	*ZCCHC14*	DOWN
Chr1	C/T	*CDK11A* (DOWN)	rs61777471	ENST00000356200.7	Novel	eQTL, sQTL	*PAX5*	DOWN
Chr1	T/C	*CDK11A* (DOWN)	rs74378830	ENST00000356200.7	Novel	mQTL	*ZFX*	DOWN
Chr11	T/G	*STARD10* (DOWN)	rs72964856	ENST00000537351.5	AD, AMD	eQTL, mQTL, sQTL	*ARAP1*	DOWN
Chr10	C/T	*KIAA1217* (UP)	rs41279872	ENST00000376462.5	AMD, AD	eQTL	*SOX5*	UP
Chr18	C/A	*CABLES1* (UP)	rs748717	ENST00000400473.6	PD, AD, AMD	eQTL	*TCF7L2*	DOWN

Note: PD—Parkinson’s disease; AD—Alzheimer’s disease, AMD—age-related macular degeneration (reported in GWAS studies).

**Table 2:** Effect of variants on miRNA binding and their targets.

Variant Gene/DEG (Expression)	miRNA	miRNA Expression	Targets	Target Expression	The Function of Target Genes
*HOXA10* (DOWN), *HOXD9* (DOWN)	Mir-29-3p	UP	*COL4A2*, *KLF4*, *ITGB1*, *COL1A2*	Down	Vasculature development
*KLC1* (DOWN)	miR-34a-5p	UP	*ERBB2*, *SRC*, *GAS1*, *PDGFR*	Down	Response to growth factor
*SEMA6A* (DOWN)	miR-124	UP	*ITGB1*, *PTBP1*, *MTDH*, *GNAI3*, *KLF6*	DOWN	Kinase signaling, angiogenesis
*HOXA9* (DOWN), *HOXA7* (DOWN)	miR-196a-5p	UP	*RANBP9*, *NOTCH2*, *SRRT*, *TRAP1*	DOWN	ERK1/2 cascade, regulation of gene expression
*PRKCI* (UP), *DLC1* (UP), *TRIM37* (UP)	miR-146a-5p	DOWN	*SOX2*, *ERBB4*, *BCLAF1*, *RAC1*	UP	Positive regulation of MAPK cascade, cellular component biogenesis

## Data Availability

Not applicable.

## Supplementary Materials

The following supporting information can be downloaded at: https://www.mdpi.com/article/10.3390/genes14091801/s1, Figure S1: Workflow for identifying the variants and differentially expressed genes/transcripts. Figure S2: Percentage of variants involved in different Quantitative Trait Loci. Figure S3: Identification of essential genes through single gene deletion analysis. Figure S4: Function interaction network of variant genes, differentially expressed genes and transcription factors. Figure S5: Number of differentially expressed genes and variant genes in different biological processes. Table S1: Dataset used for meta data analysis. Table S2: Genes identified using DTE/ DTU analysis. Table S3: Variants that affect miRNA binding and its expression in HD. Table S4: Importance of essential genes and their metabolic flux. Table S5: Gene interactions between DEGs, variant genes and transcription factors of hub genes.
