# Supplementary material for: Investigating Neuron Degeneration in Huntington’s Disease Using RNA-Seq Based Transcriptome Study
Source: Genes (Basel). 2023 Sep 14;14(9):1801. doi: 10.3390/genes14091801 (PMC10530489; doi:10.3390/genes14091801)
Supplement: Supplementary file 1 [file genes-14-01801-s001.zip › genes-2533764-supplementary.pdf]

Supplementary Information

Investigating neuron degeneration in Huntington's disease using RNA-seq based transcriptome study

Nela Pragathi Sneha[1], S. Akila Parvathy Dharshini[1], Y-h. Taguchi[2] and M. Michael Gromiha[1,*]

[1]Department of Biotechnology, Bhupat and Jyoti Mehta School of Biosciences, Indian Institute of Technology Madras, Chennai 600036, Tamilnadu, India.

[2]Department of Physics, Chuo University, Kasuga, Bunkyo-ku, Tokyo 112-8551, Japan.

[*]corresponding author

Tel: +91-2257-4138

Fax: +91-2257-4102

E-mail: gromiha@iitm.ac.in

Supplementary Table S1: Dataset used for meta data analysis

Samples	Count	Age	Post-Mortem Interval (Hours)
Controls	7	42-64	6-24
HD_samples	7	44-69	0-22

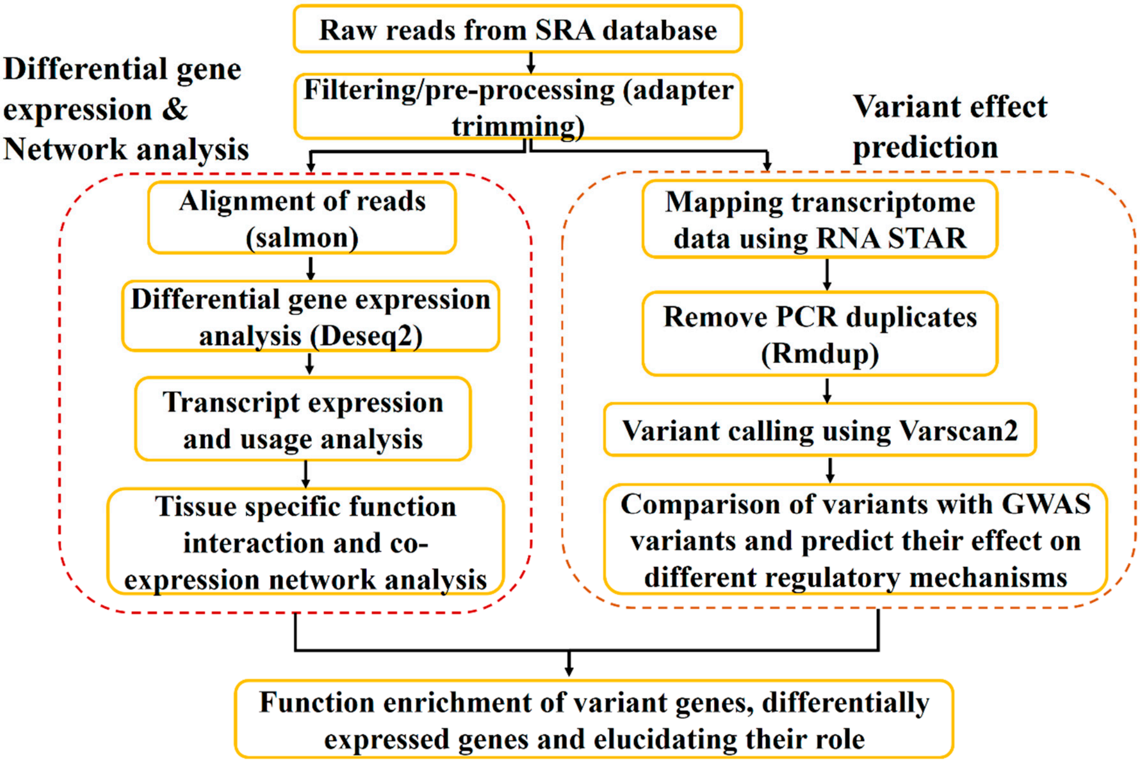

Supplementary Figure S1: Workflow for identifying the variants and differentially expressed genes/transcripts

Supplementary Table S2: Genes identified using DTE/ DTU analysis

Gene ID	Gene Name	Gene q-value	Number of transcripts	Number of significant transcripts
ENSG00000175287	*PHYHD1*	0.014434	10	1
ENSG00000120833	*SOCS2*	0.03452	8	1
ENSG00000100722	*ZC3H14*	0.020912	8	1
ENSG00000100519	*PSMC6*	1.11E-10	7	1
ENSG00000088836	*SLC4A11*	0.007169	7	1
ENSG00000167196	*FBXO22*	9.54E-06	7	1
ENSG00000144857	*BOC*	0.002836	7	1
ENSG00000169220	*RGS14*	0.020116	7	1
ENSG00000111203	*ITFG2*	0.024131	7	1
ENSG00000110077	*MS4A6A*	4.09E-11	6	2
ENSG00000104953	*TLE6*	0.00146	6	1
ENSG00000114857	*NKTR*	0.001498	6	1
ENSG00000235220	*HLA-F*	0.00554	6	1
ENSG00000100600	*LGMN*	3.88E-05	6	1
ENSG00000141076	*UTP4*	0.000273	6	1
ENSG00000128274	*A4GALT*	0.017088	6	1
ENSG00000166526	*ZNF3*	0.031697	6	1
ENSG00000267060	*PTGES3L*	0.000787	6	1
ENSG00000140153	*WDR20*	0.000649	6	1
ENSG00000164597	*COG5*	0.017088	5	1
ENSG00000136826	*KLF4*	4.04E-05	5	1
ENSG00000149781	*FERMT3*	0.000455	5	1
ENSG00000215126	*ZNG1F*	2.03E-05	5	1
ENSG00000183484	*GPR132*	0.007169	5	1
ENSG00000234651	*BAG6*	0.043997	5	1
ENSG00000130649	*CYP2E1*	0.003332	5	1
ENSG00000114062	*UBE3A*	0.000665	5	1
ENSG00000160218	*TRAPPC10*	2.99E-05	5	1
ENSG00000087303	*NID2*	0.049472	5	1
ENSG00000132141	*CCT6B*	0.033065	5	1
ENSG00000104870	*FCGRT*	0.004871	5	1
ENSG00000111452	*ADGRD1*	0.000799	5	1
ENSG00000104129	*DNAJC17*	8.03E-05	5	2
ENSG00000161526	*SAP30BP*	0.01653	5	1
ENSG00000150687	*PRSS23*	0.001452	5	1
ENSG00000168824	*NSG1*	1.50E-10	5	1
ENSG00000138442	*WDR12*	0.000891	5	1
ENSG00000185988	*PLK5*	0.028369	5	1

ENSG00000060138	*YBX3*	0.025774	5	1
ENSG00000009830	*POMT2*	0.00024	5	1
ENSG00000078487	*ZCWPW1*	0.029922	5	1
ENSG00000108231	*LGI1*	0.01283	5	1
ENSG00000115556	*PLCD4*	0.004436	5	1
ENSG00000129646	*QRICH2*	0.024131	5	1
ENSG00000088876	*ZNF343*	0.019295	5	1
ENSG00000106070	*GRB10*	0.034105	5	1
ENSG00000079459	*FDFT1*	0.007753	5	1
ENSG00000167699	*GLOD4*	0.008841	5	1
ENSG00000122687	*MRM2*	0.031947	5	1
ENSG00000143373	*ZNF687*	0.042807	5	1
ENSG00000133067	*LGR6*	0.010462	4	1
ENSG00000173085	*COQ2*	5.20E-05	4	1
ENSG00000153551	*CMTM7*	0.000511	4	1
ENSG00000196684	*HSH2D*	0.004314	4	1
ENSG00000100526	*CDKN3*	0.003846	4	1
ENSG00000206376	*EHMT2*	0.001657	4	2
ENSG00000106123	*EPHB6*	4.71E-05	4	1
ENSG00000276887	*TTYH1*	0.039631	4	2
ENSG00000109171	*SLAIN2*	2.21E-05	4	1
ENSG00000157303	*SUSD3*	0.029384	4	1
ENSG00000182378	*PLCXD1*	0.012999	4	1
ENSG00000023228	*NDUFS1*	0.00219	4	1
ENSG00000150459	*SAP18*	0.008408	4	1
ENSG00000151789	*ZNF385D*	0.018551	4	1
ENSG00000187243	*MAGED4B*	0.02358	4	1
ENSG00000101146	*RAE1*	0.039398	4	1
ENSG00000177000	*MTHFR*	0.028369	4	1
ENSG00000131778	*CHD1L*	0.03092	4	1
ENSG00000156097	*GPR61*	0.007957	4	1
ENSG00000172551	*MUCL1*	0.038699	4	1
ENSG00000112280	*COL9A1*	0.000344	4	1
ENSG00000156531	*PHF6*	0.013047	4	1
ENSG00000147364	*FBXO25*	0.024465	4	1
ENSG00000136560	*TANK*	0.025721	4	1
ENSG00000148339	*SLC25A25*	0.001416	4	1
ENSG00000262860	*LSM14A*	0.000283	4	1
ENSG00000143106	*PSMA5*	0.011914	4	1
ENSG00000128253	*RFPL2*	0.047869	4	1
ENSG00000106853	*PTGR1*	0.001342	4	1
ENSG00000157837	*SPPL3*	0.005656	4	1
ENSG00000170035	*UBE2E3*	0.026186	4	1

ENSG00000072518	*MARK2*	0.017807	4	1
ENSG00000144744	*UBA3*	0.026984	4	1
ENSG00000185104	*FAF1*	0.013047	4	1
ENSG00000232045	*EHMT2*	0.000455	3	3
ENSG00000124256	*ZBP1*	0.004675	3	1
ENSG00000164744	*SUN3*	0.027889	3	1
ENSG00000228299	*HLA-C*	0.000795	3	1
ENSG00000273622	*CDC42EP5*	0.007582	3	2
ENSG00000281385	*AP2A2*	0.008295	3	1
ENSG00000276725	*CEP170*	0.012527	3	1
ENSG00000240230	*COX19*	5.01E-10	3	2
ENSG00000150054	*MPP7*	0.000107	3	2
ENSG00000125850	*OVOL2*	0.000625	3	1
ENSG00000145220	*LYAR*	3.50E-07	3	2
ENSG00000134940	*ACRV1*	0.003478	3	1
ENSG00000181856	*SLC2A4*	0.014434	3	1
ENSG00000236014	*VPS52*	0.003712	3	2
ENSG00000231129	*ABCF1*	0.007183	3	2
ENSG00000132792	*CTNNBL1*	0.001312	3	1
ENSG00000151131	*NOPCHAP1*	0.000283	3	1
ENSG00000278229	*RPS17*	0.033215	3	2
ENSG00000130699	*TAF4*	0.047869	3	1
ENSG00000186174	*BCL9L*	0.001099	3	1
ENSG00000102144	*PGK1*	0.000787	3	1
ENSG00000274081	*PUF60*	0.000595	3	1
ENSG00000103707	*MTFMT*	9.54E-06	3	1
ENSG00000181450	*ZNF678*	0.047908	3	1
ENSG00000165731	*RET*	0.029181	3	1
ENSG00000277273	*CDK7*	0.030087	3	1
ENSG00000167680	*SEMA6B*	0.03039	3	1
ENSG00000198168	*SVIP*	0.02941	3	1
ENSG00000120158	*RCL1*	0.037738	3	1
ENSG00000065665	*SEC61A2*	0.007487	3	1
ENSG00000278318	*ZNF229*	0.024131	3	1
ENSG00000115561	*CHMP3*	0.04319	3	1
ENSG00000170043	*TRAPPC1*	0.000283	3	1
ENSG00000169181	*GSG1L*	0.035077	3	1
ENSG00000137996	*RTCA*	0.031947	3	2
ENSG00000168495	*POLR3D*	0.000787	3	1
ENSG00000129083	*COPB1*	0.006458	3	1
ENSG00000173409	*ARV1*	0.000795	3	1
ENSG00000162735	*PEX19*	0.028196	3	1
ENSG00000239961	*LILRA4*	0.013484	2	2

ENSG00000213931	*HBE1*	0.00037	2	2
ENSG00000230475	*ABHD16A*	0.004137	2	2
ENSG00000237071	*TRIM27*	0.01394	2	2
ENSG00000228570	*NUTM2E*	0.038699	2	2
ENSG00000212866	*HSPA1B*	0.00554	2	2
ENSG00000162843	*WDR64*	0.038869	2	2
ENSG00000226882	*GNL1*	0.007774	2	2
ENSG00000196993	*NPIPB9*	0.008408	2	2
ENSG00000230596	*GPAA1P2*	0.012904	2	2
ENSG00000223532	*HLA-B*	0.028369	2	2
ENSG00000213648	*SULT1A4*	0.025579	2	2
ENSG00000290457	NA	0.017969	2	2
ENSG00000214274	*ANG*	0.028938	2	2
ENSG00000129757	*CDKN1C*	0.047367	2	2
ENSG00000278728	*CNTNAP2*	3.78E-07	2	2
ENSG00000149089	*APIP*	0.007228	2	2
ENSG00000214026	*MRPL23*	0.008408	2	2
ENSG00000170915	*PAQR8*	0.012809	2	2
ENSG00000274332	*LRRC37A2*	0.023126	2	2
ENSG00000169635	*HIC2*	0.018651	2	2
ENSG00000273673	NA	0.028369	2	2
ENSG00000078237	*TIGAR*	0.013047	2	2
ENSG00000170893	*TRH*	0.038802	2	2
ENSG00000188916	*INSYN2A*	0.038747	2	2
ENSG00000224740	*FLOT1*	0.038699	2	2
ENSG00000128285	*MCHR1*	0.000103	2	2
ENSG00000076003	*MCM6*	0.003846	2	2
ENSG00000276802	*HERC2*	0.002292	2	2
ENSG00000155962	*CLIC2*	0.028369	2	2
ENSG00000130720	*FIBCD1*	0.033065	2	2
ENSG00000213753	*CENPBD2P*	0.000663	2	2
ENSG00000205423	*CNEP1R1*	0.007487	2	2
ENSG00000130703	*OSBPL2*	0.023126	2	2
ENSG00000139291	*TMEM19*	0.042593	2	2
ENSG00000112852	*PCDHB2*	0.010462	2	2
ENSG00000198783	*ZNF830*	0.033863	2	2
ENSG00000125851	*PCSK2*	0.03092	2	2
ENSG00000265681	*RPL17*	0.001342	2	2
ENSG00000160214	*RRP1*	0.032229	2	2
ENSG00000004059	*ARF5*	0.018977	2	2
ENSG00000134153	*EMC7*	7.89E-05	2	2
ENSG00000131016	*AKAP12*	0.01056	2	2
ENSG00000163655	*GMPS*	0.002232	2	2

ENSG00000198242	*RPL23A*	0.026173	2	2
ENSG00000137198	*GMPR*	0.02223	2	2
ENSG00000169217	*CD2BP2*	0.028092	2	2
ENSG00000146006	*LRRTM2*	0.02223	2	2
ENSG00000134508	*CABLES1*	0.008408	2	2
ENSG00000100902	*PSMA6*	0.025213	2	2
ENSG00000165704	*HPRT1*	0.013047	2	2
ENSG00000213463	*SYNJ2BP*	0.020489	2	2

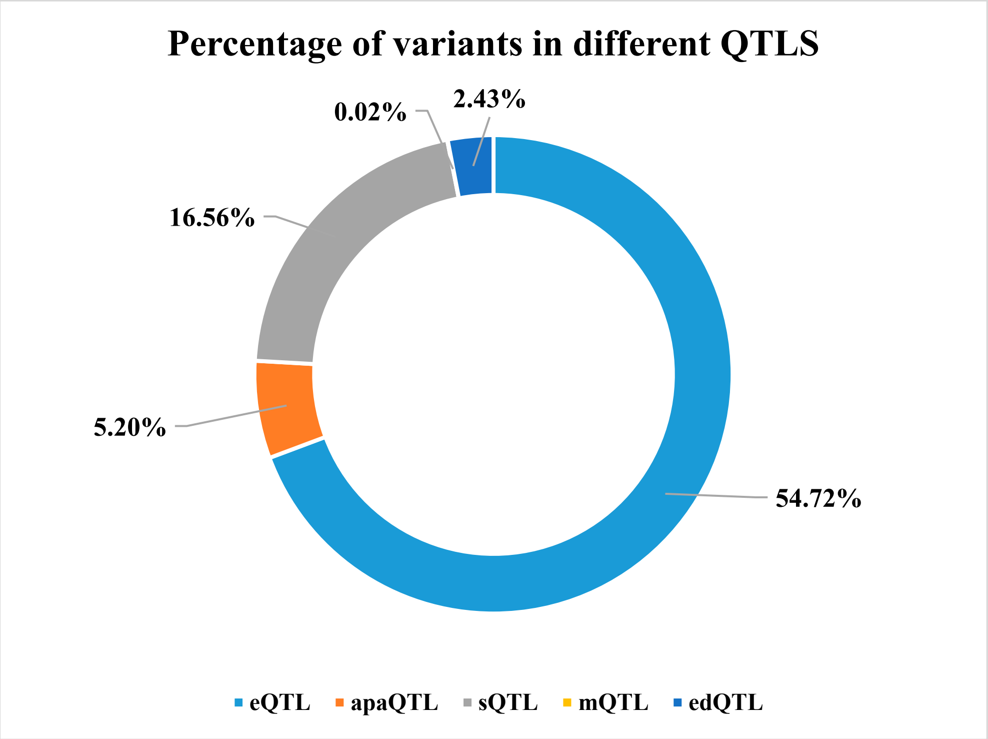

Supplementary Figure S2: Percentage of variants involved in different Quantitative Trait Loci

Supplementary Table S3: Variants that affect miRNA binding and its expression in HD.

**Chromosome**	**Alt/Ref**	**Gene**	**SNP**	**GWAS/novel SNP**	**Affected miRNA**	**miRNA expression**	**miRNA target gene/s**	**Expression of gene**
Chr16	G/C	*SMPD3*	rs12927738	Novel	miR-29-3p	UP	*COL4A2, KLF4, ITGB1, COL1A2*	DOWN
Chr5	T/C	*EFNA5*	rs252682	AD, PD	miR-29-3p	UP	*COL4A2, KLF4, ITGB1, COL1A2*	DOWN
Chr1	C/T	*KLC1*	rs4652791	AMD	miR-34a-5p	UP	*ERBB2, SRC, GAS1, PDGFR*	DOWN
Chr5	G/T	*SEMA6A*	rs258012	Novel	miR-124	UP	*ITGB1, PTBP1, MTDH, GNAI3*	DOWN
Chr7	G/T	*HDAC9*	rs2520362	PD,AMD	miR-196a-5p	UP	*RANBP9, NOTCH2, SRRT, TRAP1*	DOWN
Chr2	C/T	*IGFBP5*	rs11575134	PD,AMD	miR-146a-5p	DOWN	*SOX2, ERBB4, BCLAF1, RAC1*	UP
Chr6	T/G	*BCLAF1*	rs180998508	Novel	miR-146a-5p	DOWN	*SOX2, ERBB4, BCLAF1, RAC1*	UP

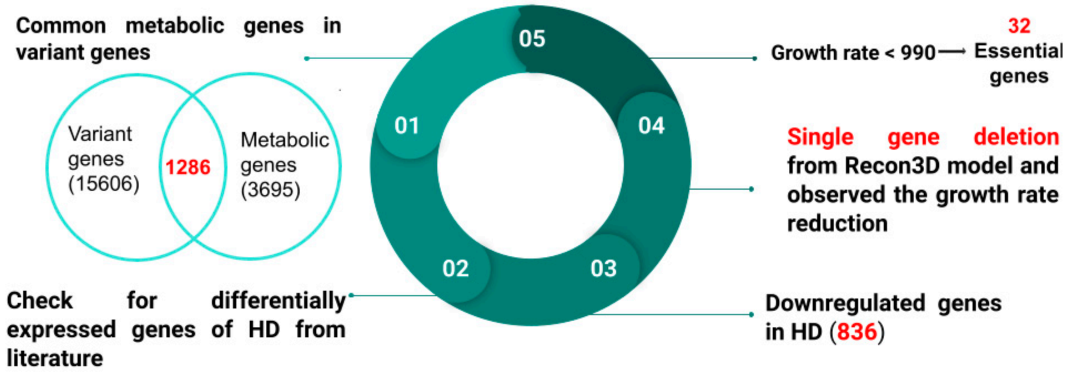

Supplementary Figure S3: Identification of essential genes through single gene deletion analysis

Supplementary Table S4: Importance of essential genes and the their metabolic flux

GENE	Number of reactions deleted through gene deletion	Metabolic flux	Importance of the gene
SLC25A10	20	789.3583	Knockdown of this gene causes defects in mitochondrial respiration[108]
CMPK1	40	826.2042	Eliminating this gene will inhibit proliferation and increase apoptosis [109]
RRM1	6	879.9701	Knockout of this gene will trigger apoptosis and inhibit growth significantly [110]
GBE1	1	880.7468	Knockdown of *GBE1* makes it more susceptible in neuron degeneration [111]
PYGL	1	904.0637	Removal of this gene from the system effects the metabolic pathways and cause glycogen storage disease [112]

ACSL1	21	916.7597	Knockout of this gene causes major metabolic defects like mitochondria based long chain FA degradation [113]
GPI	3	919.3579	Removal of GPI gene induces cell proliferation inhibition [114]
DERA	1	925.6295	Knockdown of this gene causes stress within mitochondria which starts a cascade of events for energy crisis in brain [115]
TALDO1	1	932.2939	*TALDO1* is a crucial gene involving in metabolic pathways and its removal can cause major impact on the metabolic system [116]
MCCC2	1	943.0063	Knockout study on *MCCC2* showed that it induces cell proliferation [117]
PGLS	2	945.7904	*PGLS* is crucial and a knockdown study in glioblastoma is performed on PGLS and it is found that NADPH and GSH neurotransmitter are reduced [118]
DMAC2L	1	947.3364	Removal of *DMAC2L* results in reduced carbohydrate metabolism and in turn restricts motility/ movement. [119]
GUK1	3	951.0216	*GUK1* is a major participant of GTP and dGTP metabolism and its removal can impact majorly on metabolic pathways that aid in energy supply for brain [120][121]
PGD	3	951.9599	Knockout of this gene will cause increased cell stress and expansion of endoplasmic reticulum volume leading to energy catastrophe [122]
PFKL	5	956.2257	*PFKL* gene inhibits glycolysis , a crucial mechanism in energy pumping when it is knocked down.[123]
DBI	21	958.2383	*DBI* is mostly found to be expressed in astrocytes and its removal will cause

			alterations in GABA signalling that is crucial in brain [124]
HMGCR	4	959.3648	Removal of *HMGCR* will cause ER stress in the cell which is a sign for energy insufficiency[125]
BCAT2	3	963.2047	*BCAT2* gene removal will effect mitochondrial respiration, oxygen consumption rate of cells [126]
CPT2	46	964.122	Knockout study on *BCAT2* effects the beta oxidation [127]
SDHB	2	965.545	*CPT2* is a crucial metabolic gene and reduced complex 2 activity is observed when removed in zebrafish [128]
CTH	2	970.6769	*CTH* is top gene producing H_2S in blood vessels and its knockdown results in brain vasculature development [129]
CPT1A	12	970.9974	Knockdown of *CPT1A* caused metabolic disturbances in the cell [130]
NADSYN1	3	976.0198	*NADSYN1* is an essential gene due to its role in neovascularization and its impairment can cause major loss of function in blood vessel morphogenesis [131]
COX8A	2	976.4597	Loss of *COX8A* gene will result in epilepsy and leigh like syndrome – movement related disorders [132]
COX7A2	2	976.4597	Knock down of *COX7A2* will lead to locomotor defect [133]
COX4I1	2	976.4597	*COX4I1* is a crucial gene and reduced neuronal network activity is observed [134]
COX7B	2	976.4597	Altered CNS activity is observed when *COX7B* is knocked out [135]

COX6C	2	976.4597	Removal of *COX6C* will lead to developmental death in cells which makes it crucial [136]
COX5A	2	976.4597	Knock down of this gene induces STRESS and lead to autophagy [137]
SLC37A4	3	981.487	*SLC37A4* is a key activator of autophagy so, its absence will create major impact on metabolic pathways of brain [138]
DPYD	2	982.7158	*DPYD* produces inhibitory neurotransmitters which are crucial for functioning of neuronal population and knockdown will have a great impact on them [139]
PTDSS1	1	986.7217	Removal of gene *PTDSS1* may lead to reduced neuron growth/ axon extension [140]

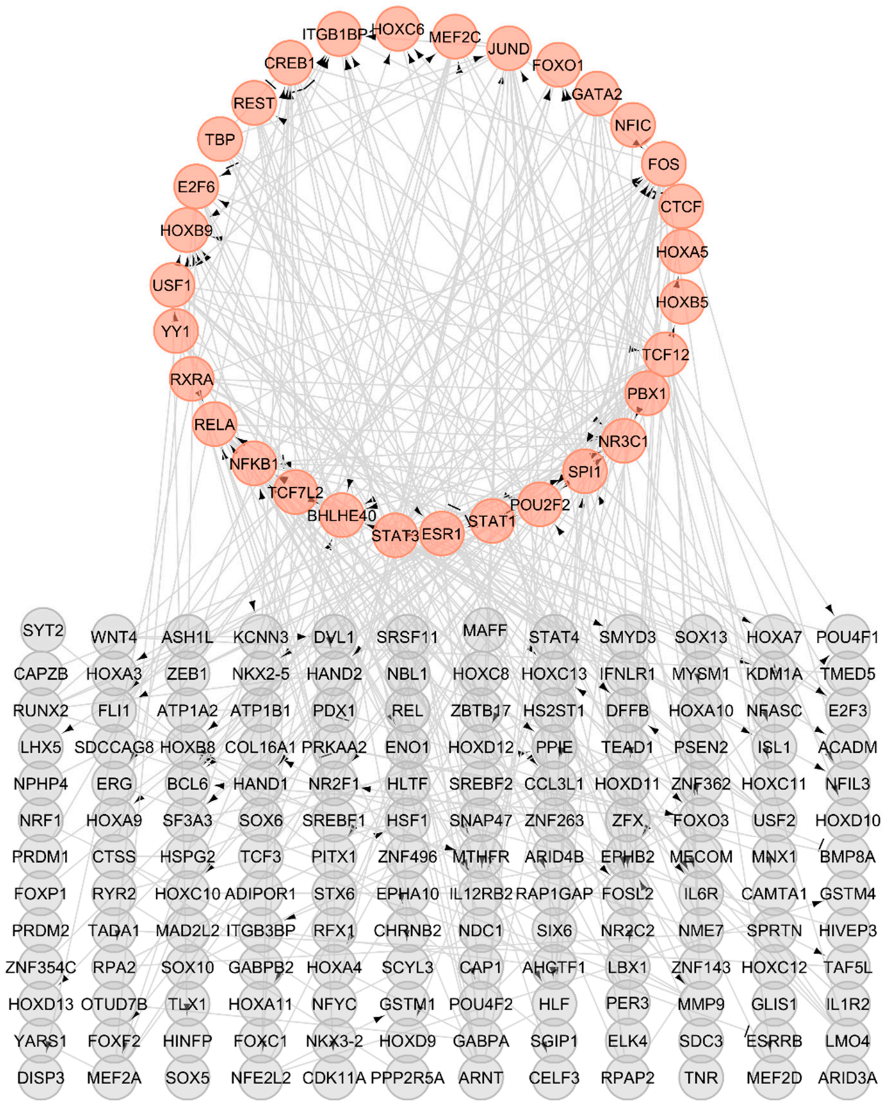

Supplementary Figure S4: Function interaction network of variant genes, differentially expressed genes and transcription factors. Orange color nodes represent hub genes based on network properties like degree, betweenness centrality. Grey nodes represent non-hub genes of FI network.

Supplementary Table S5: Gene interactions between DEGs, variant genes and transcription factors of hub genes

GENE1	FI Direction	GENE2	FI Score	FI Annotation
HOXA5	←	*TCF12*	1	expression regulated by
NR3C1	←	*SPI1*	1	expression regulated by; reaction
NR3C1	←|	*PBX1*	1	activated by; inhibit
NR3C1	←	*RXRA*	1	activated by
NR3C1	←	*RELA*	1	activate; activated by; complex; inhibit
NR3C1	←|	*TBP*	1	activated by; inhibit
NR3C1	←|	*RUNX2*	1	activated by; inhibit
HOXC6	←	*SPI1*	1	expression regulated by
HOXC6	←	*ZEB1*	1	expression regulated by
HOXC6	←	*TCF12*	1	expression regulated by
ESR1	-	*LMO4*	1	complex
ESR1	-|	*POU4F2*	1	complex; inhibit
ESR1	-	*USF2*	1	complex
ESR1	-	*KDM1A*	1	complex
ESR1	→	*POU4F1*	1	activate; complex
ESR1	→	*HOXA10*	1	expression regulates
ESR1	|→	*NR3C1*	1	activate; inhibited by
ESR1	→	*OTUD7B*	1	expression regulates
ESR1	|→	*NFIC*	1	activate; inhibited by
ESR1	-	*TBP*	1	complex
ESR1	←	*USF1*	1	complex; expression regulated by
ESR1	→	*JUND*	1	complex; expression regulates; reaction
JUND	→	*TEAD1*	1	expression regulates
JUND	→	*NR2F1*	1	expression regulates
JUND	→	*SPI1*	1	expression regulates
JUND	→	*POU2F2*	1	expression regulates
JUND	→	*PRDM1*	1	expression regulates
JUND	←	*TCF12*	1	expression regulated by; expression regulates
JUND	→	*MEF2C*	1	expression regulates
JUND	←	*TBP*	1	expression regulated by
JUND	→	*STAT3*	1	expression regulates
JUND	→	*STAT1*	1	expression regulates
JUND	←	*SREBF1*	1	expression regulated by
JUND	→	*SREBF2*	1	expression regulates
GATA2	→	*SPI1*	1	complex; expression regulates
GATA2	→	*ZEB1*	1	expression regulates
GATA2	-	*TCF3*	1	complex
GATA2	→	*HLF*	1	expression regulates
GATA2	→	*NR3C1*	1	expression regulates

GATA2	→	*TCF12*	1	complex; expression regulates
CREB1	-	*TCF12*	1	complex
CREB1	|→	*NR3C1*	1	activate; expression regulates; inhibited by
CREB1	|→	*ZBTB17*	1	activate; inhibited by
CREB1	→	*KCNN3*	1	expression regulates
CREB1	|→	*REST*	1	activate; inhibited by
CREB1	→	*TBP*	1	expression regulates; reaction
CREB1	-	*NFE2L2*	1	complex
CREB1	→	*STAT3*	1	expression regulates; reaction
CREB1	-	*MEF2D*	1	complex; reaction
CREB1	←	*NFKB1*	1	expression regulated by; reaction
TCF12	←	*USF1*	1	expression regulated by
SPI1	←	*ZNF263*	1	expression regulated by
SPI1	←	*STAT3*	1	expression regulated by
SPI1	→	*ZEB1*	1	expression regulates
SPI1	-	*TBP*	1	complex
SPI1	→	*USF1*	1	expression regulates
SPI1	←	*USF2*	1	expression regulated by
TBP	-	*ZNF143*	1	complex
TBP	|→	*ZBTB17*	1	activate; inhibited by
MEF2C	←	*ZNF263*	1	expression regulated by
MEF2C	-	*TCF3*	1	complex
MEF2C	←	*USF1*	1	expression regulated by
MEF2C	-	*NKX2-5*	1	complex; reaction
RELA	←|	*SREBF1*	1	activated by; inhibited
RELA	←|	*SREBF2*	1	activated by; inhibit
RELA	←	*STAT4*	1	activated by
RELA	←|	*RXRA*	1	activated by; inhibited
YY1	→	*ZNF143*	1	expression regulates
YY1	→	*ZBTB17*	1	expression regulates
HOXB9	←	*MAFF*	1	expression regulated by
HOXB9	←	*USF1*	1	expression regulated by
HOXB9	←	*JUND*	1	expression regulated by
HOXB9	←	*SREBF1*	1	expression regulated by
HOXB9	←	*TCF7L2*	1	expression regulated by
HOXB9	←	*TCF12*	1	expression regulated by
HOXB9	←	*RXRA*	1	expression regulated by
CTCF	→	*NFIL3*	1	expression regulates
CTCF	-	*RXRA*	1	complex
CTCF	→	*ITGB1BP1*	1	expression regulates
CTCF	-	*PBX1*	1	complex
CTCF	→	*TCF12*	1	expression regulates
CTCF	→	*SPI1*	1	expression regulates
CTCF	→	*FLI1*	1	expression regulates
CTCF	→	*STAT3*	1	expression regulates

CTCF	→	*HOXA5*	1	expression regulates
ITGB1BP1	←	*USF1*	1	expression regulated by
ITGB1BP1	←	*ZNF263*	1	expression regulated by
ITGB1BP1	←	*MAFF*	1	expression regulated by
ITGB1BP1	←	*RXRA*	1	expression regulated by
ITGB1BP1	←	*SPI1*	1	expression regulated by
ITGB1BP1	←	*YY1*	1	expression regulated by
ITGB1BP1	←	*JUND*	1	expression regulated by
NFKB1	→	*REL*	1	complex; expression regulates
NFKB1	←|	*SREBF2*	1	activated by; inhibit
NFKB1	←|	*SREBF1*	1	activated by; inhibit
NFKB1	←	*SPI1*	1	expression regulated by
NFKB1	→	*STAT1*	1	expression regulates
NFKB1	←	*STAT4*	1	activated by
NFKB1	|→	*NR3C1*	1	activate; complex; expression regulates; inhibited by; input
NFKB1	→	*POU2F2*	1	activate; expression regulates
NFIC	→	*NR3C1*	1	expression regulates
BHLHE40	→	*HOXB9*	1	expression regulates
BHLHE40	←	*TCF7L2*	1	expression regulated by
BHLHE40	←	*STAT3*	1	expression regulated by
BHLHE40	←	*NR3C1*	1	expression regulated by
BHLHE40	←	*TCF12*	1	expression regulated by
BHLHE40	←	*MEF2C*	1	expression regulated by
BHLHE40	←	*MEF2A*	1	expression regulated by
USF1	←	*YY1*	1	expression regulated by
USF1	-	*USF2*	1	complex; input
REST	←	*TCF12*	1	activated by; expression regulates; inhibit
FOS	→	*HLF*	1	expression regulates
FOS	-	*TCF7L2*	1	complex; input
FOS	-	*GATA2*	1	complex; input; reaction
FOS	|→	*NFIC*	1	activate; inhibited by
FOS	|→	*STAT4*	1	activate; inhibited by; reaction
FOS	→	*HLTF*	1	expression regulates
FOS	→	*ZNF143*	1	expression regulates
FOS	→	*RFX1*	1	expression regulates
FOS	|→	*NR3C1*	1	activate; complex; inhibited by; input; reaction
FOS	→	*FOSL2*	1	expression regulates
FOS	←	*JUND*	1	complex; expression regulated by
FOXO1	←	*LMO4*	1	activated by
FOXO1	-	*NFYC*	1	complex
FOXO1	←	*IL6R*	1	activated by
FOXO1	-	*NR3C1*	1	complex; reaction
E2F6	→	*TCF12*	1	expression regulates

E2F6	→	*HLF*	1	expression regulates
E2F6	→	*ZEB1*	1	expression regulates
E2F6	←	*NR3C1*	1	expression regulated by
E2F6	←	*JUND*	1	expression regulated by
E2F6	→	*ENO1*	1	expression regulates
E2F6	→	*MEF2A*	1	expression regulates
E2F6	→	*POU2F2*	1	expression regulates
E2F6	→	*PER3*	1	expression regulates
HOXB5	←	*TCF12*	1	expression regulated by
POU4F2	←	*REST*	1	expression regulated by

FI: Functional Interaction

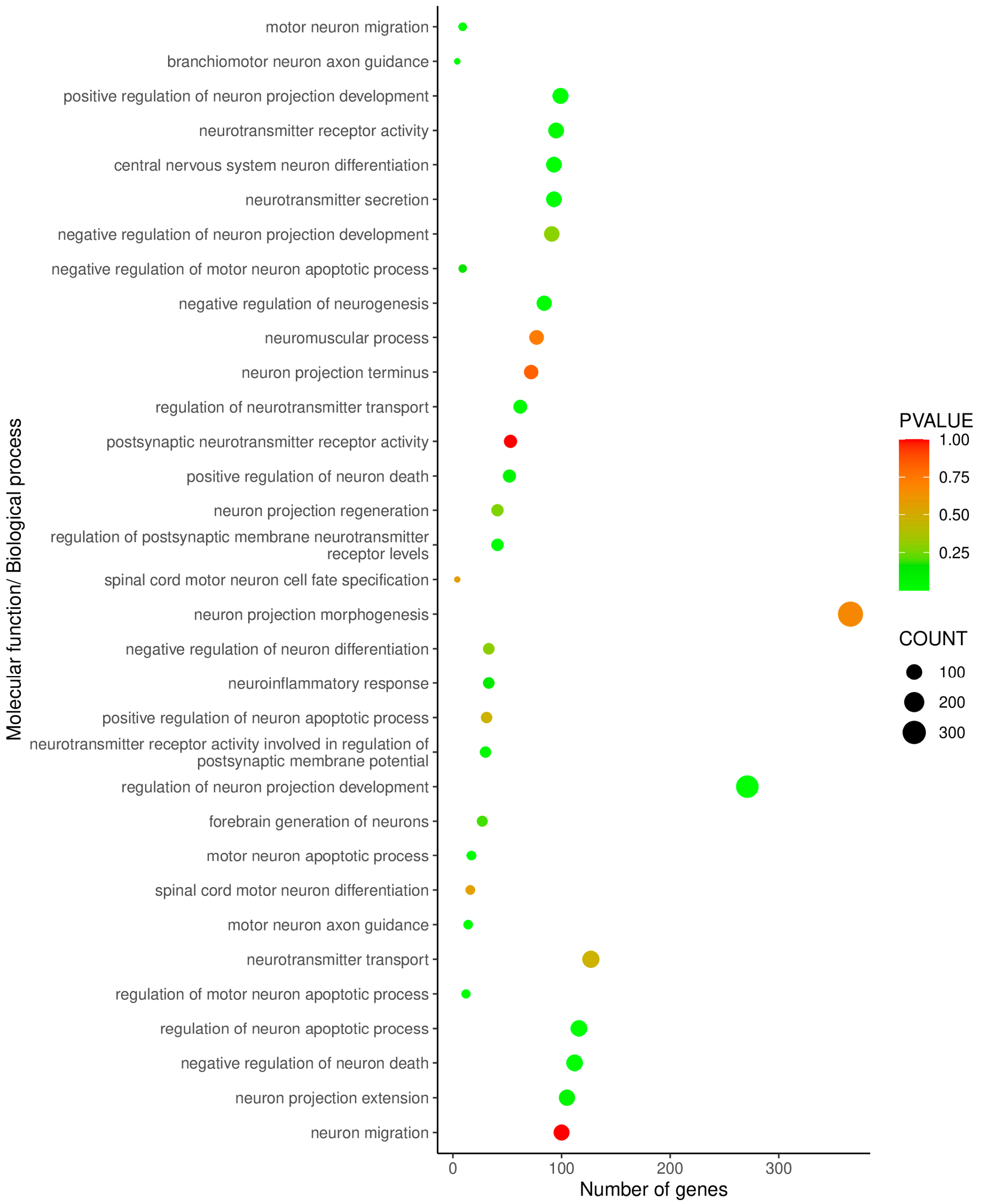

Supplementary Figure S5: Number of differentially expressed genes and variant genes in different biological processes
